# Fluconazole Analogs and Derivatives: An Overview of Synthesis, Chemical Transformations, and Biological Activity

**DOI:** 10.3390/molecules29122855

**Published:** 2024-06-15

**Authors:** Michał Janowski, Oleg M. Demchuk, Monika Wujec

**Affiliations:** 1 Doctoral School, Medical University of Lublin, Chodzki 7, 20-093 Lublin, Poland; jjjmichal1@gmail.com; 2 Faculty of Medicine, The John Paul II Catholic University of Lublin, Konstantynow 1J, 20-708 Lublin, Poland; 3 Department of Organic Chemistry, Faculty of Pharmacy, Medical University of Lublin, Chodzki 4a, 20-089 Lublin, Poland

**Keywords:** fluconazole, synthesis, chemical modifications, biological activity, antifungal activity

## Abstract

Fluconazole (2-(2,4-difluorophenyl)-1,3-bis(1*H*-1,2,4-triazol-1-yl)propan-2-ol), which was patented in 1981 and introduced for commercial use in 1988, is a widely utilized antifungal drug whose mechanism of action involves inhibition of the activity of 14-α lanosterol demethylase. Its safety and effectiveness have established it as one of the most frequently employed antifungal agents. Resistance to azole antifungal drugs is becoming more common. It may be related to a mutation of the gene encoding the enzyme. To address this issue, molecules with modifications in three main regions of fluconazole, namely the hydroxyl group, the aromatic ring, and the 1,2,4-triazole rings, have been synthesized in an attempt to create more potent antifungal drugs. These modifications aim at enhancing the effectiveness against microorganisms and improving pharmacokinetic parameters and safety profiles of the synthesized compounds. The present review explores the synthesis of fluconazole derivatives, accompanied by insights into the results of biological studies evaluating the therapeutic effects of these compounds.

## 1. Introduction

In the face of the increasing number of fungal infection cases, the imperative to refine antifungal therapies has become a pressing issue in medicine [1]. In recent years, there has been an alarming rise in microbial resistance to existing antifungal drugs, posing a significant challenge to effective fungal infection treatment. The diversity of mechanisms developed by microorganisms to evade the action of drugs often renders existing therapies ineffective, making infections difficult to control [2,3]. Given this threat, there is an imperative to develop new derivatives of antifungal drugs that not only counteract the development of resistance by pathogens but also exhibit strong anti-pathogenic effects and enhance the safety of the therapy. Furthermore, improving the pharmacokinetic parameters of new substances may contribute to increasing their effectiveness and precise delivery to the site of infection, a key element of effective antifungal therapy [4].

Fluconazole is an azole antifungal drug that acts by selectively inhibiting the enzyme 14α-demethylase, which is crucial for the biosynthesis of ergosterol in fungal cells. Disruption of ergosterol production weakens the cell membranes of fungi, thereby disrupting osmotic homeostasis and inhibiting their growth. Fluconazole differs from other azoles due to its specific selectivity towards the fungal cytochrome P450 [5]. Its mechanism of action focused on 14α-demethylase makes it an effective drug, especially in combating infections caused by *Candida* spp. and *Cryptococcus neoformans* [6,7].

A study using global pharmaceutical sales data from 2008–2018 across 65 countries found that fluconazole was one of the most commonly used antifungal agents globally, along with itraconazole and terbinafine. Although we can observe a decrease in the use of antifungal drugs in high-income countries, global consumption of systemic antifungal agents increased during this period, with an overall annual growth rate of 6.2% from 2008 to 2018, due to a significant rise in their use in middle-income countries.

Fluconazole is highly effective and safe, as evidenced by its use in neonatal therapy [8]. Given its safety in therapy, it is often widely used as a drug that promotes the development of microbial resistance. Since the patent protecting fluconazole expired in 2005, its structural motif has become very attractive for new drug design. Synthesizing new, effective fluconazole derivatives enables pharmaceutical companies to patent these new structures, leading to the introduction of original and profitable medications, especially since fluconazole itself can now be used in generic products [9]. In this review, we will focus on analyzing recent advancements in the synthesis of fluconazole derivatives. Our goal is to present progress in this field and emphasize the need for further research aimed at developing more effective, safe, and precise antifungal therapies.

**Scheme 1:**
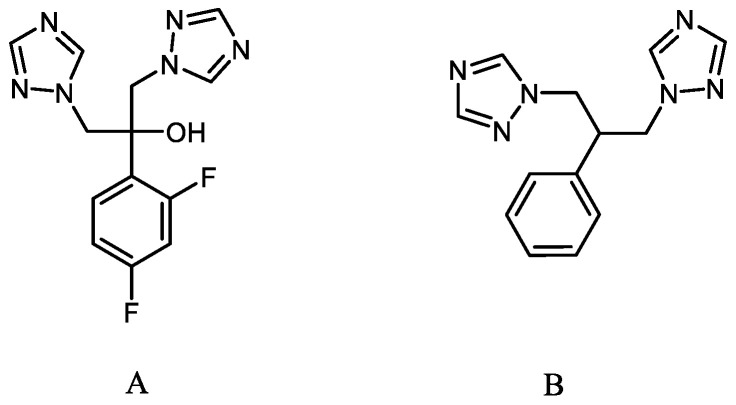
Structural formula of fluconazole (**A**) and its core (**B**) used for literature search.

We have analyzed the literature looking for the fluconazole structural motif as presented in Scheme 1. The time of original publication (both patents and journals articles) was not limited, but former reviews that only partially covered the topic were cited but not critically analyzed.

## 2. Syntheses of Fluconazole

**Scheme 2:**
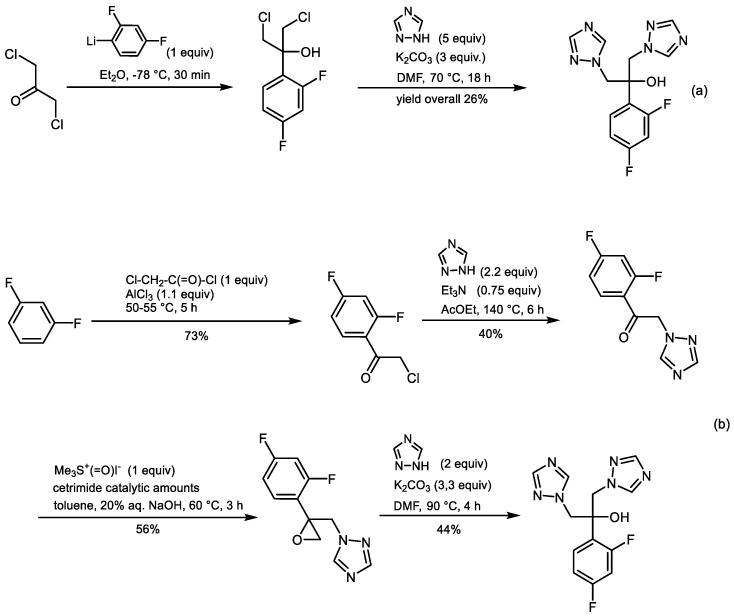
Original approaches to fluconazole synthesis based on: the Snieckus direct *ortho*-lithiation (**a**), the Friedel–Crafts acylation (**b**).

There are several approaches to fluconazole synthesis. The originals developed by Pfizer were based on the Snieckus direct *ortho*-lithiation of *meta* difluorobenzene followed by quenching the formed anion with 1,3-dichloroacetone and *N*-alkylation of 1,2,4-triazole, as presented in Scheme 2a [10]. The same patent protects the alternative approach based on the Friedel–Crafts acylation of difluorobenzene Scheme 2b.

**Scheme 3:**
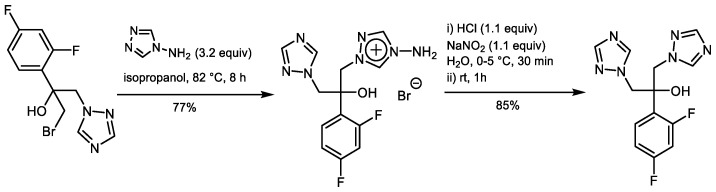
Improved regioselective synthesis of fluconazole through avoidance of the oxirane intermediate.

Many minor improvements of those methods were covered by other patents. For example, the synthesis method described in the 1991 patent by Industriale Chimica Srl protected the process throughout the synthesis of the amino fluconazole derivative (Scheme 3, [11]). This method solves the issue of the regioselectivity of the oxirane ring opening.

**Scheme 4:**
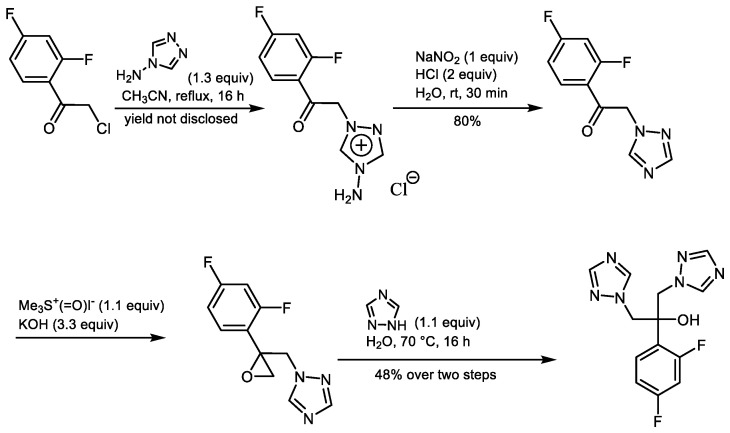
Improved synthesis of fluconazole through the utilization of the aminotriazole and oxirane steps.

The modification of the above approach was protected by the Development Center of Biotechnology of Taiwan with a patent published in 1996 [12]. In this strategy, the authors declare that the selectivity of the synthesis could be improved by utilizing aminotriazole in the first condensation step, as presented in Scheme 4.

**Scheme 5:**
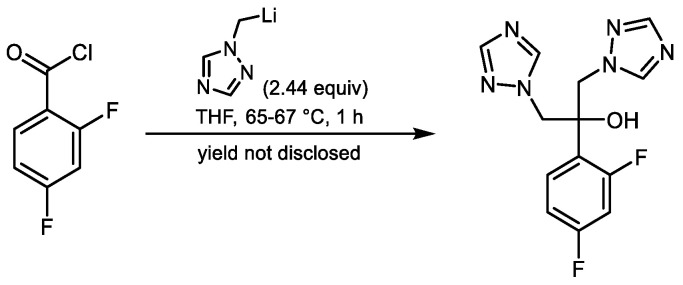
Synthesis of fluconazole using [(1*H*-1,2,4-triazol-1-yl)methyl]lithium.

The Centro Genesis Para la Investigacion S.L. protected other approaches, based on the direct lithiation of methylotriazole with *n*-BuLi, followed by condensation with 2,4-difluorobenzoyl chloride (Scheme 5, [13]).

**Scheme 6:**
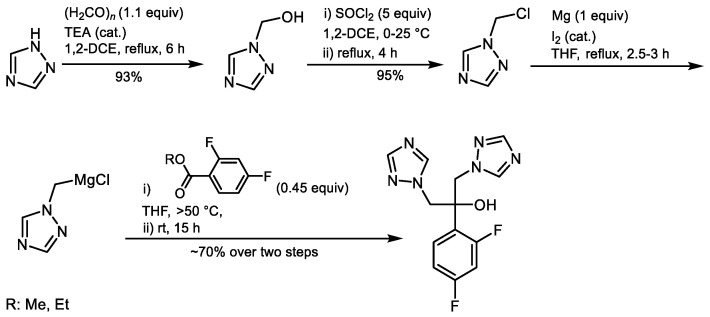
Approaches based on the utilization of [(1*H*-1,2,4-triazol-1-yl)methyl)]magnesium chloride.

Zhejiang Genebest Pharmaceutical Co., Ltd. (Hang zhou, China) in 2012 [14], proposed the replacement of the lithium derivative with corresponding Grignard reagent and 2,4-difluorobenzoyl chloride with a corresponding ester, as presented in Scheme 6. A few years later, the Board of Regents of Oklahoma State University made the same suggestion [15].

**Scheme 7:**
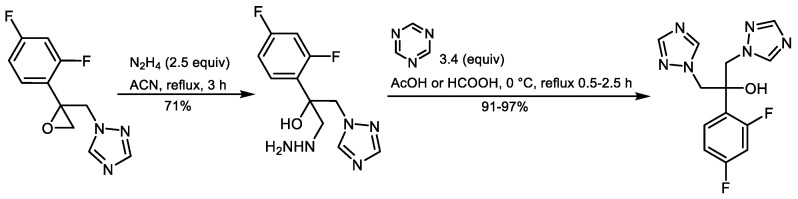
*s*-Triazine based synthesis of fluconazole.

Fluconazole is usually obtained through the use of triazole derivatives; nevertheless, there are other synthetic approaches. Centro Genesis Para La Investigacion, S.L. and Krka also patented the triazole ring-closure approach (Scheme 7, [16]). At the key step of the sequence used, a hydrazine derivative underwent annulation in the reaction with *s*-triazine carried out in acidic conditions.

**Scheme 8:**
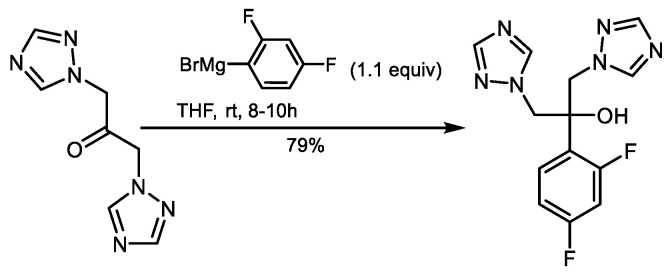
Synthesis of fluconazole following the Grignard strategy.

Another method of preparation of fluconazole involved altering the sequence of the originally proposed reactions. In the first step, dichloroacetone was condensed with triazole, followed by its reaction with 2,4-difluorophenylmagnesium bromide (Scheme 8, [17]).

**Scheme 9:**
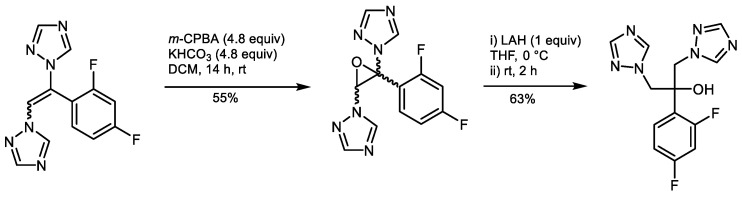
2,4-Difluorostyrene pathway to fluconazole.

The unusual approach was protected in 1997 with a patent created by Apotex Inc. (Toronto, Canada) (Scheme 9, [18]). It was proposed to use bis-triazole substituted 2,4-difluorostyrene.

**Scheme 10:**
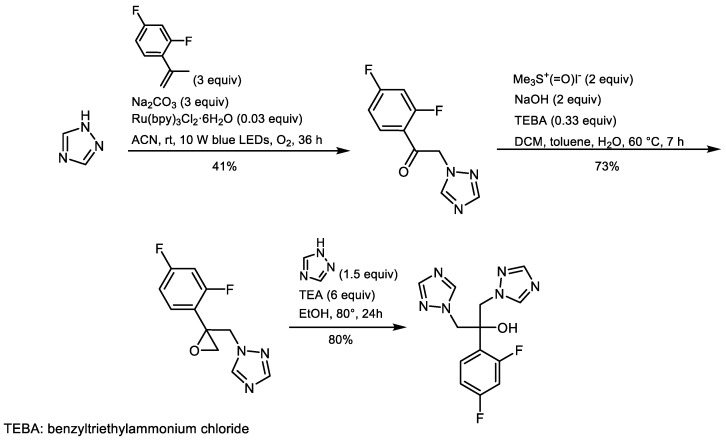
Visible-light-induced synthesis of fluconazole via the regioselective radical oxo-amination step.

Recently, the synthesis of a key intermediate of the original approach was achieved through visible light-induced regioselective radical oxo-amination of alkenes, including 2,4-difluoro-α-methylstyrene with O_2_ as the oxygen atom source [19]. Ruthenium catalyst was employed to mediate the double activation of C-H and C-C bonds, as illustrated in Scheme 10.

**Scheme 11:**
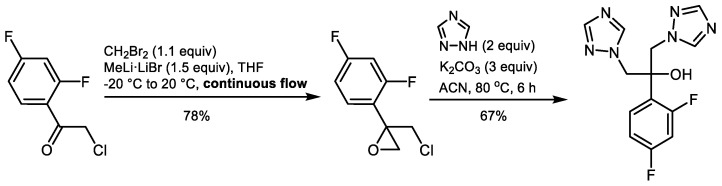
Semi-continuous flow synthesis of the key intermediate epichlorohydrin, used in the synthesis of fluconazole.

One more variation of the original approach was published in 2019 (Scheme 11, [20]). The study was based on the continuous flow synthesis of epoxide, starting from ketone and using in situ-generated bromomethyl lithium. Modifying the process to include the epichlorohydrin intermediate allows the transformation to occur in a semi-continuous flow mode.

The Grignard reagent-based synthesis of fluconazole presented by Korwar S. et al. [21] is also methodologically interesting. The authors of the publication utilized Knochel’s so called turbo-Grignard reagents to form 2,4-difluorophenylmagnesium bromide, which was employed instead of 2,4-difluorophenyllithium, and conducted the synthesis as presented in Scheme 2a; however, it was performed in continuous flow mode.

The syntheses of fluconazole presented in this chapter do not encompass all the patented examples, but rather clarify the methodologies employed. Approaches that have limited scientific value, yet serve to address IP manufacturing concerns, were omitted. The syntheses of fluconazole derivatives presented below are, in most cases, based on these general approaches.

## 3. Fluconazole Analogs with an Unsubstituted Hydroxy Group

### 3.1. Isotope-Substituted Fluconazole

**Scheme 12:**
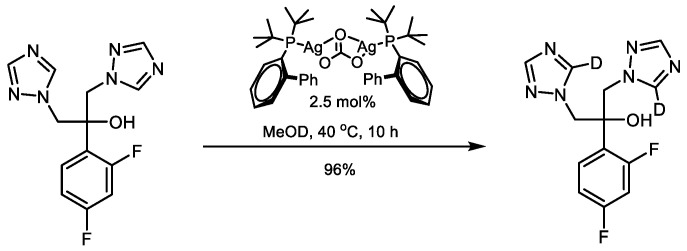
Site-selective silver-catalyzed C-H bond deuteration of fluconazole.

Site-selective silver-catalyzed C-H bond deuteration of fluconazole was presented by Adrian Tlahuext-Aca et al. in 2021 [22]. It has been shown that silver complexes of the well-established C,P-ligand JohnPhos [23] along with some other ligands used in cross-coupling reactions [24] readily mediate the H/D exchange between fluconazole (and other five-membered heteroaromatics) and MeOH-d_1_, as presented in Scheme 12. The publication thoroughly discussed the mechanistic aspects of the reaction used. The results are important for the synthesis of deuterated pharmaceuticals with improved pharmacodynamic and pharmacokinetic properties as well as for internal standards for LC/MS quantification.

**Scheme 13:**
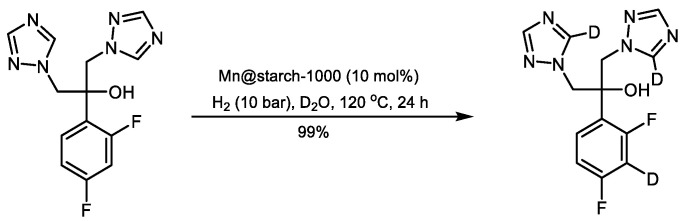
Mn@Starch-NP mediated protium/deuterium exchange in fluconazole.

In 2022, F. Bourriquen et al. presented the synthesis of deuterium-labeled fluconazole obtained in an exchange reaction mediated by manganese core–shell nanoparticles (Scheme 13, [25]). The deuteration mediated by Mn@Starch-NPs was more demanding and required higher catalyst loading and a higher temperature applied for a longer time. Thus, tris-deuterium substituted fluconazole was obtained in a good yield. Additionally, other electron-rich(hetero)arenes and some anilines also undergo this reaction.

**Scheme 14:**
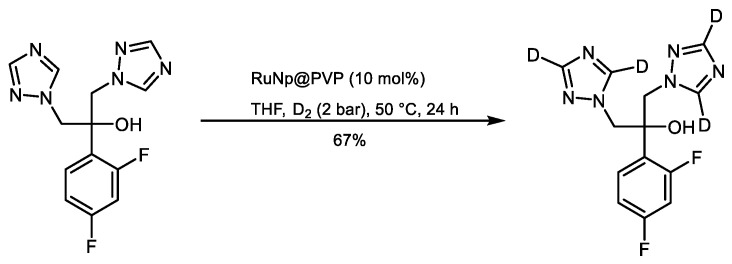
RuNp@PVP mediated the H/D replacement in the fluconazole molecule.

The quantum-chemical mechanistic investigations of the hydrogen isotope exchange mediated by Ru nanocatalysts were reported in 2020 [26]. These findings provide insights into the nature of chemical processes responsible for the isotope exchange, explaining the observed regioselectivity and kinetics of the H/D replacement. The theoretical results obtained were then utilized to achieve the synthesis of fully deuterated triazole rings in fluconazole. The reaction was run in THF with utilization of 10 mol% of RuNp@PVP catalyst at 50 °C under 2 bar of D_2_ for 24 h (Scheme 14). Other *N*-heterocycles and some carbocyclic compounds also undergo this deuteration reaction.

**Scheme 15:**
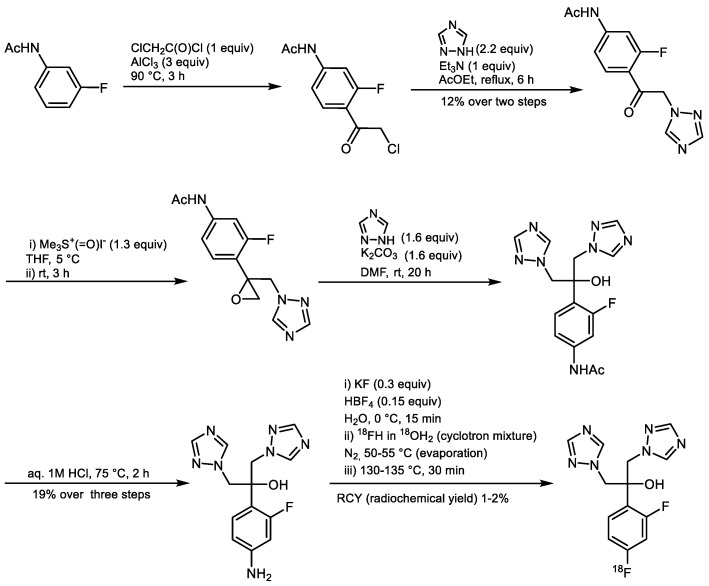
Synthesis of ^18^F_-_labeled fluconazole.

The preparation of fluorine-18-labeled fluconazole is important for positron emission tomography studies. Such a derivative was obtained by applying the modified synthesis of fluconazole (Scheme 15, [27]). Biological studies indicate that the radiopharmaceutic maintains the antifungal behavior of the unlabeled drug and exhibits a biodistribution similar to that of ^14^C-labeled fluconazole.

Unfortunately, we were unable to localize the synthesis of ^14^C-labeled fluconazole in patent or research publications.

### 3.2. Fluconazole Analogs with Different Substituents on the Carboaromatic Ring and/or a C-Substituted Methylene Linkage

**Scheme 16:**
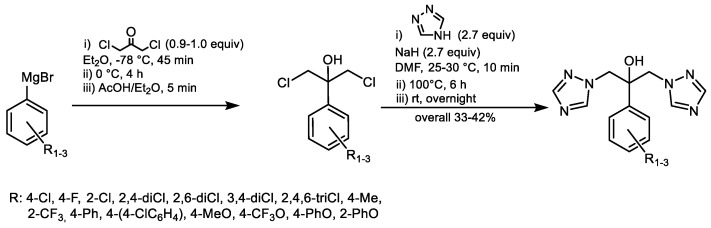
Synthesis of fluconazole analogs using Grignard reagents.

In 1982, Imperial Chemical Industries issued a patent for the method for synthesizing analogs of fluconazole (Scheme 16, [28]) to be used as antifungal agents in agriculture. Modifications were made to the structure of the carboaromatic ring. The substituents were introduced into the structure of the corresponding Grignard reagent, which was utilized in the synthesis of fluconazole derivatives according to the usual approach. Biological tests were conducted to assess the inhibition of growth and the inhibition of fungal infections by these compounds in cultivated plants. A compound with two chlorine atoms (2,4-diCl) exhibited the most potent antifungal activity while not significantly inhibiting plant growth.

**Scheme 17:**
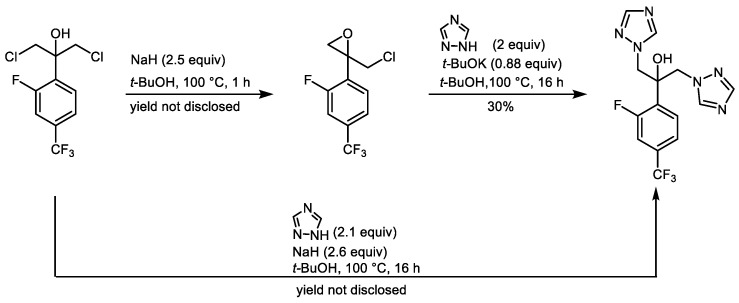
Analog of fluconazole synthesis featuring a trifluoromethyl group at position C-4 of phenyl.

In 1985, the same company patented similar syntheses of fluconazole analogs (Scheme 17, [29]), aiming at potential applications in medicine and veterinary. Two competitive pathways were claimed, but the direct comparison of the methods was not proposed. These analogs featured a trifluoromethyl group at position 4 of the carboaromatic ring. Parameters for the Minimum Effective Dose (MED) were determined in vivo for *Candida albicans* and *Trichophyton mentagrophytes* in mice, establishing a MED of 5 mg/kg. The MED value was determined to be 10 mg/kg for rabbits and 25 mg/kg for rats. It was determined that the compound is not teratogenic at doses lower than those toxic to the mother, based on experiments with rabbits.

**Scheme 18:**
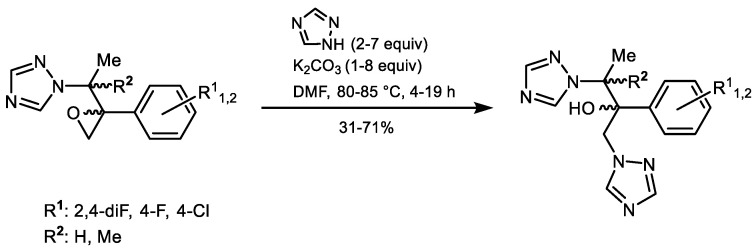
Synthesis of fluconazole analogs substituted at one of the methylene moieties with a methyl group.

Aiming at potential antifungal activity for use in medicine and veterinary applications, in 1984, Schering Corporation (Kenilworth, NJ, USA) (Scheme 18, [30]) created a patent that included the synthesis of some fluconazole non-symmetric analogs by branching one of the methylene connections to the carbinolic carbon together with modifications in the aromatic ring substitution.

In vivo studies were conducted by determining the PD50, specifying the dose at which 50% of mice infected with *C. albicans* were preserved from death. The mice were infected with *C. albicans* and the PD value of the most active compound was 0.1 mg/kg of the drug. Additionally, Mean Survival Time (MST) studies were conducted, comparing the compound to ketoconazole. The most active compound, namely *rac*-2-(2,4-difluorophenyl)-1,3-bis(1*H*-1,2,4-triazol-1-yl)butan-2-ol (Scheme 18, R*^2^*: H), extended MST by 20 days, compared to ketoconazole.

**Scheme 19:**
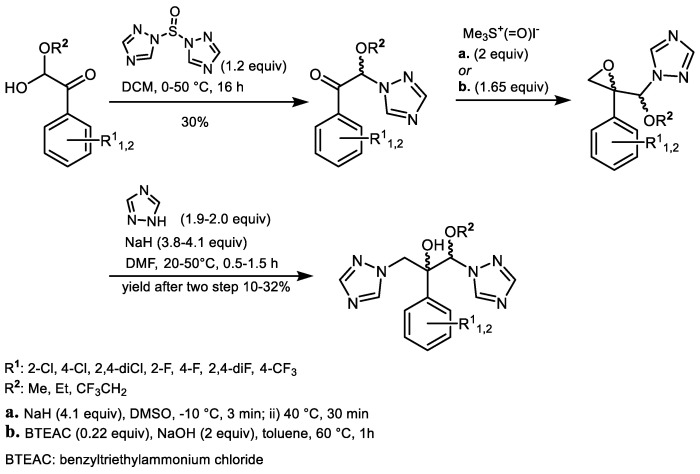
Synthesis of fluconazole analogs with alkoxy substitution at the methylene moiety.

The patent granted to Shionogi Seiyaku Kabushiki Kaisha in 1987 described a method for the synthesis of new chiral analogs of fluconazole due to the presence of alkoxy substituents of one of the methylene linkages and modifications in the carboaromatic ring region. Two variations of the method were proposed. The synthesis route in which NaH was utilized at the epoxide formation stage led to the formation of diastereoisomer A (as named by the authors), whereas the synthesis pathway with BTEAC was used to obtain diastereoisomer B (Scheme 19, [31]). Although the stereo configurations of diastereoisomers have not been established and it is only known that they have different polarities, one could assume that depend on the reaction conditions *syn*- or *anti*- isomers (or their mixture) of the target fluconazole analog were isolated after a chromatographic followed by crystallization purification. The diastereoisomers A was formed under the conditions described in Scheme 19a with lower yields than diastereoisomers B formed under the conditions described in Scheme 19b, usually in a mixture with A.

According to the authors, these compounds could be used in the treatment of fungal infections in humans and animals. In vitro studies were conducted to determine the Minimal Elimination Concentration (MEC), assessing this parameter based on the inhibition of pseudohyphal formation in *C. albicans*. For the most active compound (Scheme 19, R^1^: 2,4-diCl, R^2^: MeO) the MEC was determined to be 0.31 μg/mL. In vivo survival studies were conducted on a mouse model infected with *C. albicans* for 5 days, administering the substances at a dose of 50 mg/kg. In those studies, the anti-*C. albicans* activity of diastereoisomer B of 2-(2,4-difluorophenyl)-1-methoxy-1,3-di(1*H*-1,2,4-triazol-1-yl)propan-2-ol (Scheme 19 R^1^: 2,4-diF R^2^: Me) was two-fold higher than the activity of diastereoisomer A. Some of the obtained analogs of fluconazole demonstrated 100% survival of infected mice.

**Scheme 20:**
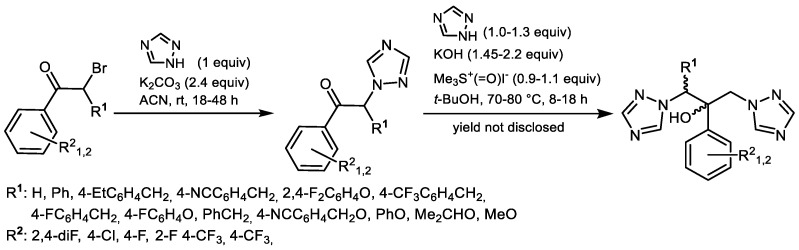
Synthesis of fluconazole derivatives containing modifications at the methylene moiety and in the carboaromatic ring.

In 1989, Imperial Chemical Industries issued a patent for the method of synthesis of fluconazole analogs with diversified substitution at one of the methylene units and modifications in the carboaromatic ring (Scheme 20, [32]). However, the stereoconfiguration of the product formed was not elucidated. The authors expected that the compounds would exhibit aromatase inhibitory activity in vitro and in vivo, but no biological studies were reported.

**Scheme 21:**
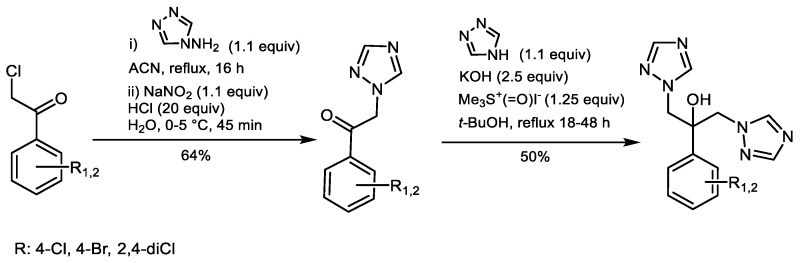
Synthesis of fluconazole analogs with halogens in the carboaromatic ring.

In 1993, A. Narayanan et al. (Scheme 21, [33]) described the synthesis of three new analogs of fluconazole with modifications in the carboaromatic ring. Two derivatives had bromine or chlorine atoms at position 4, and one derivative had chlorine atoms at positions 2 and 4. No biological activity was determined for these derivatives.

**Scheme 22:**
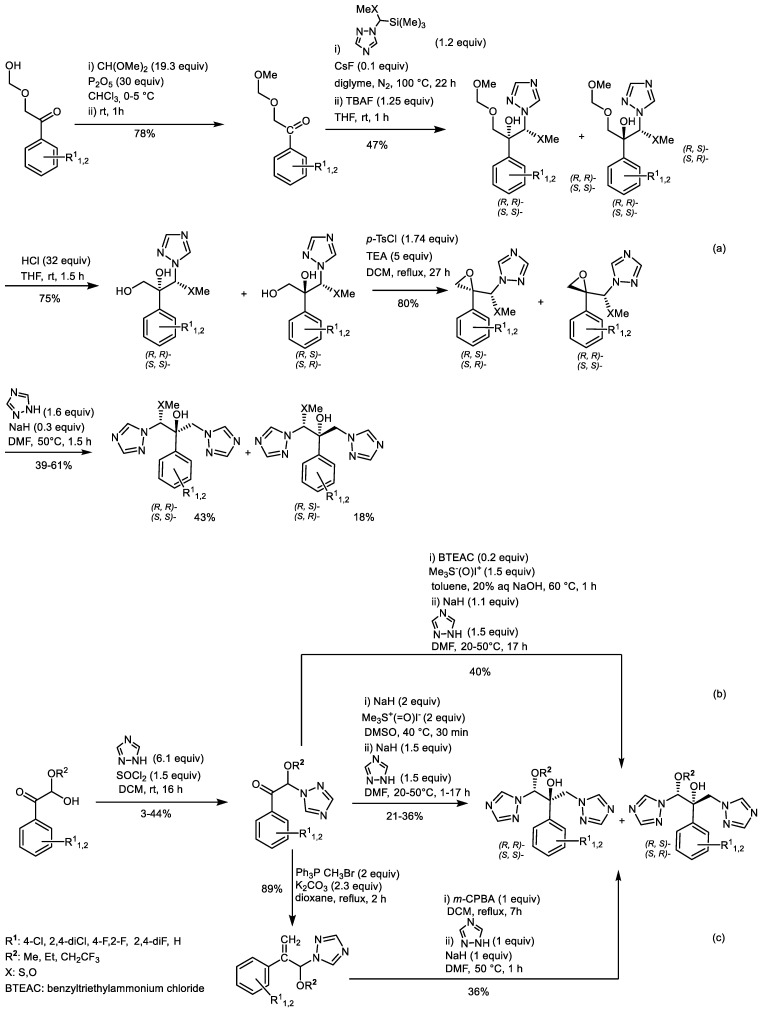
Synthesis of chiral non-racemic analogs of fluconazole via: cyclization of 1,3-diol (**a**), oxirane formation with Me_3_S(O)I (**b**), epoxidation of alkene (**c**).

In 1989, M. Ogata et al. (Scheme 22, [34]) reported the synthesis of new chiral non-racemic analogs of fluconazole with an additional substituent at the methylene moiety and modifications in the carboaromatic ring. The authors developed a new methodology in which, starting with readily available starting materials, the oxirane ring can be formed in three alternative ways: as a product of cyclization of 1,3-diol (Scheme 21a), in a reaction with Me_3_S(O)I (Scheme 22b), and via epoxidation of Wittig reaction-delivered alkene with *m*-PCBA (Scheme 22c). In fact, although the authors claimed that they obtained enantiomerically pure compounds with absolute configurations (*1R*,*2R)*- and (*1R*,*2S)*-, the analysis of the original publications allows one to assume that the racemic mixtures of diastereomers (*1R*,*2R)*-/(*1S*,*2S)*- and (*1R*,*2S)*-/(*1S*,*2R)*- were obtained instead.

In vitro antifungal activity testing was carried out, determining MIC values against *C. albicans*, *Aspergillus fumigatus*, and *Trichosporon asteroides*. No activity against *C. albicans* and *A. fumigatus* was observed at doses below 100 μg/mL. However, some compounds exhibited satisfactory activity against *T. asteroides*, with the most active compound with the absolute configuration (*1R*,*2R*)- and the following substituents R^1^: 2,4-diF, X: O, R^2^: Me showing an MIC of 0.8 μg/mL. Additionally, it also exhibited the strongest activity with a MEC of 0.31 μg/mL in the inhibition of *C. albicans* pseudomycelium formation and the most potent efficacy in in vivo studies of mouse survival, where 100% survival was determined in mice infected with *C. albicans* after 7 days upon administration of the drug orally at a dose of 50 mg/kg.

**Scheme 23:**
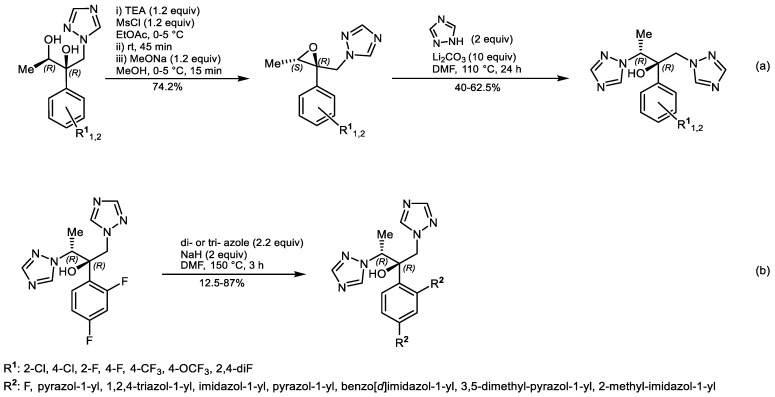
Synthesis of fluconazole analogs with a methyl group and different substituents in the carboaromatic ring via: cyclization of enantiopure 1,3-diol (**a**), defluoroamination reaction (**b**).

Takeda Chemical Industries Ltd. issued a patent in which derivatives with a methyl group in the aliphatic chain and modifications within the aromatic ring were presented (Scheme 23, [35]). It is difficult to state precisely which compounds were actually obtained, but the authors indicated that the preferable R^1^ groups were a hydrogen atom, a halogen atom, a haloalkyl group, or a haloalkoxy group. In particular, it was beneficial when one of the selected R^1^ atoms is a halogen atom and another is a hydrogen atom or a halogen atom. The halogen atom could be fluorine, chlorine, bromine, or iodine, with fluorine being preferred. The halogen atom was desirably bonded at 2- and/or 4-position(s) (Scheme 23a). Compounds possessing an azole moiety were obtained in a course of a strong base-facilitated defluoroamination reaction (Scheme 23b). The antifungal activities of the compounds were determined by measuring the diameter of the growth inhibition zone against *C. albicans* IFO 0583. The inhibition zone diameter of the most active compound was determined to be 20 mm.

**Scheme 24:**
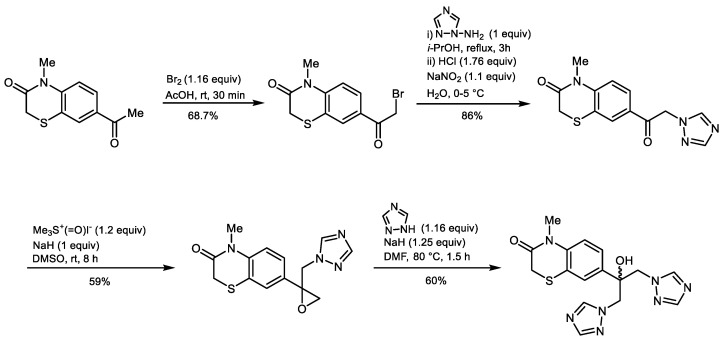
Synthesis of a fluconazole analog containing a 4*H*-benzo[*b*][1,4]thiazine unit.

In 1998, R. Fringuelli et al. (Scheme 24, [36]) presented the synthesis of a new derivative of fluconazole with a 4*H*-benzo[*b*][1,4]thiazine unit. The compound was designed to be a more effective antifungal agent. In vitro antifungal activity testing against *C. albicans* was conducted by comparing the parameter to fluconazole. The minimum inhibitory concentration (MIC) of the new derivative was >250 μg/mL versus <1 μg/mL in the case of fluconazole. In vivo studies determined median survival times (MST) in comparison to fluconazole, with the obtained compound showing an MST of 23 days, whereas fluconazole had an MST of >60 days. Additionally, colony-forming units (CFU) in the livers of infected mice were assessed three days post-inoculation, resulting in CFU = 77.8 ± 15.7 for the new compound, while the CFU value of fluconazole was equal to 0. The new compound showed no in vitro activity but exhibited some activity in in vivo studies, although said activity was lower than that of fluconazole.

**Scheme 25:**
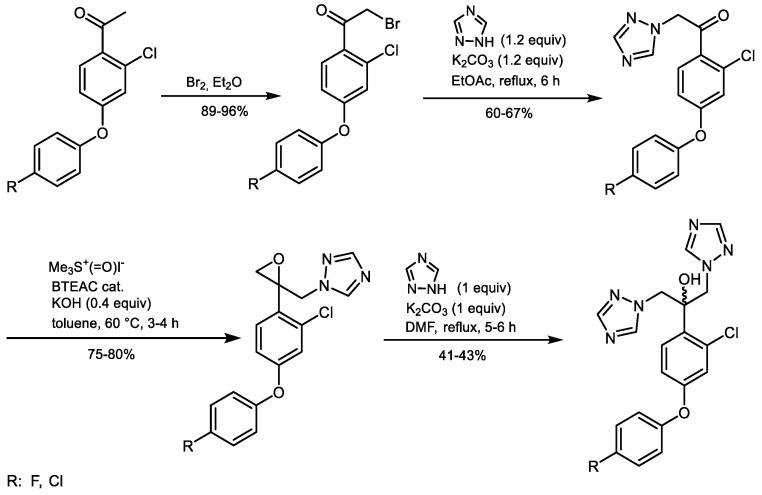
Synthesis of fluconazole analog containing a phenoxy group at the 4-position of the carboaromatic ring.

In 2009, G-P. Yu et al. (Scheme 25, [37]) reported the synthesis of two new analogs of fluconazole bearing a substituted phenoxy group at position 4 of the carboaromatic ring.

**Scheme 26:**
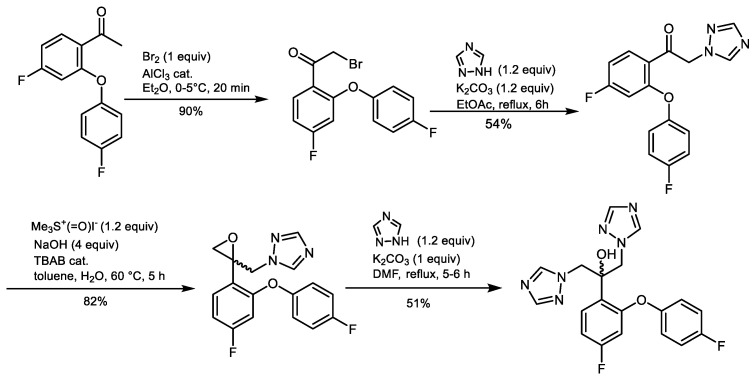
Synthesis of a 4-fluoro-2-(4-fluorophenoxy)phenyl analog of fluconazole.

These compounds were designed to possess a potential antifungal application in agriculture. Fungicidal tests against *Giberella zeae*, *Alternaria solani*, *Cercospora arachidicoa*, *Physalospora pircola*, and *Fusarium oxysporum* compared the compounds to terbuconazole and difenoconazole. The percentage inhibition of growth was determined for three substance concentrations: 50, 5, and 1 μg/mL. The 4-(4-fluorophenoxy) analog (Scheme 26, R: F). showed higher activity than the reference substances against *F. oxysporum* at a concentration of 1 μg/mL, achieving an inhibition rate of 90%. This molecule also exhibited high activity against the other strain tested. A similar synthesis of a fluconazole analog in which the fluorine atom at position 2 was replaced by a 4-fluorophenoxy group was published in 2015 by S-H. Yang et al. (Scheme 26, [38]). This compound underwent investigations of its fungicidal properties, determining the percentage of growth inhibition at a concentration of 50 μg/mL. It exhibited satisfactory properties against *G. zeae* (44.1%), *A. solani* (20.0%), *C. archidicola* (50.0%), *F. oxysporum* (38.5%), and *P. pircola* (41.2%).

**Scheme 27:**
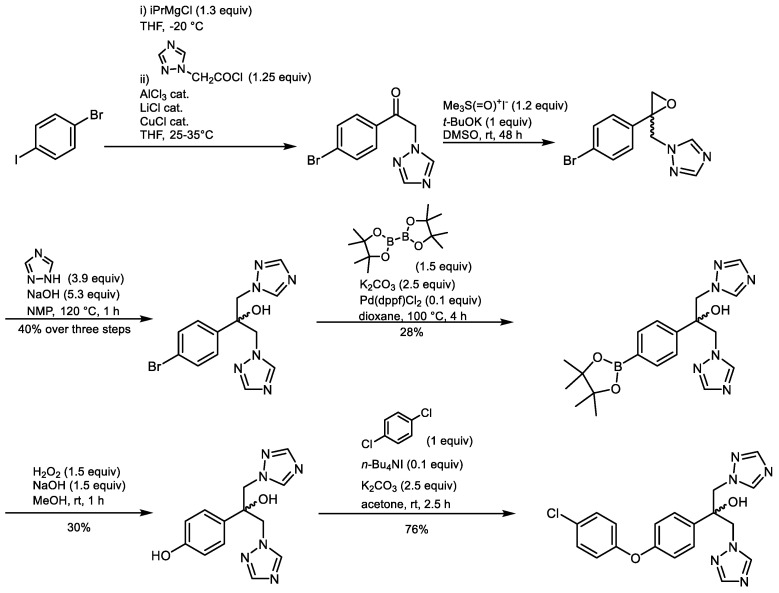
Synthesis of a fluconazole analog containing the 4-(4-chlorophenoxy)phenyl group.

In 2015, the BASF SE corporation patented a method for synthesizing a new derivative of fluconazole with modifications within the carboaromatic ring (Scheme 27, [39]). The compounds were positioned as potentially applicable antifungal agents in agriculture. However, no biological tests were conducted for these derivatives.

### 3.3. Fluconazole Analogs Containing N,C-Substituted-1,2,4-triazol-1-yl Units

**Scheme 28:**
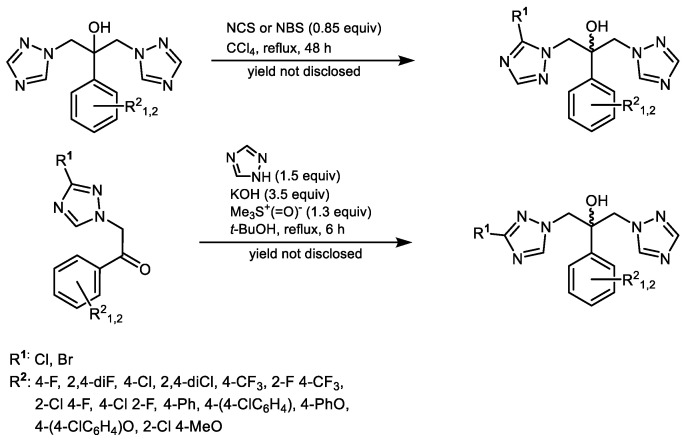
Synthesis of fluconazole derivatives substituted at position 3 of the 1,2,4-triazole ring.

In 1985, Imperial Chemical Industries patented a method for synthesizing analogs of fluconazole with modifications within the carboaromatic and 1,2,4-triazole rings (Scheme 28, [40]). The syntheses leading to the desired derivatives followed a known approach but utilized initially substituted aromatic fragments and introduced halogen substituents in the initial or final reaction steps. The synthesized compounds were intended as fungicidal agents with potential applications in medicine or agriculture. However, no biological activity tests were conducted for these compounds.

**Scheme 29:**
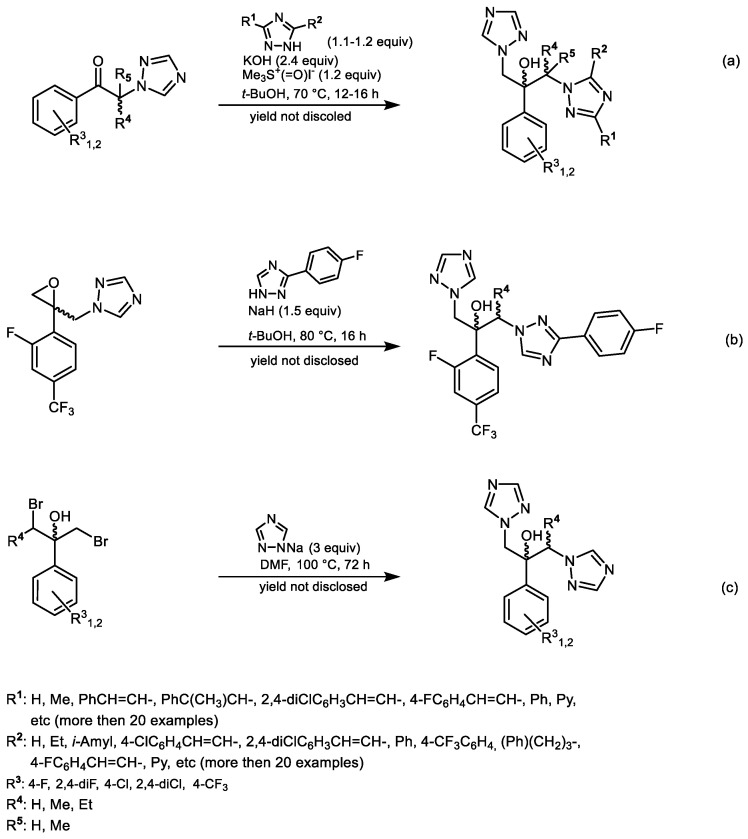
Synthesis of analogs od fluconazole *via*: acetophenones (**a**), oxirane (**b**), and dibromopropan-2-ols (**c**) key intermediates.

In 1984, Imperial Chemical Industries patented the synthesis of a large library of fluconazole analogs. These compounds featured substituents in the methylene group and in the (carbo- and hetero-) aromatic systems (Scheme 29, [41]).

The compounds were proposed as potential antifungal agents to be used in human and animal health care. In the case of one of the compounds obtained applying the oxirane pathway (R^1^: H; R^2^: 4-ClC_6_H_4_CH=CH-; R^3^: 2,4-diCl; R^4^: H), the Minimum Effective Dose (MED) was determined in a mouse model infected with *C. albicans* in the vagina and *T. mentagrophytes* on the back. The initial dose was 250 mg/kg, but this was gradually reduced to assess the minimum dose required for recovery, and the determined MED was 5 mg/kg.

**Scheme 30:**
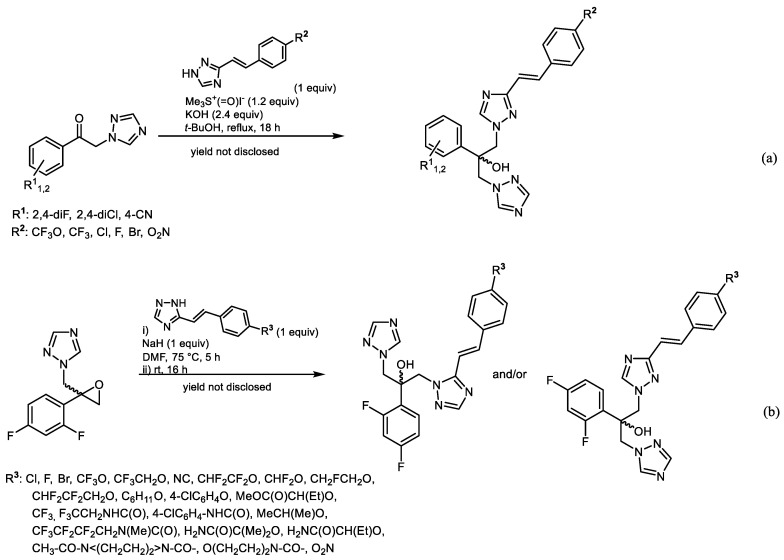
Synthesis of fluconazole analogs with an *E*-styryl- substituent at position 3 (**a**) and at positions 3 or 5 (**b**) of the 1,2,4-triazole moiety.

In 1986, Imperial Chemical Industries patented the synthesis of fluconazole analogs with modifications in the structure of the triazole ring (Scheme 30, [42]). These compounds were suggested to have potential applications in veterinary medicine or as antifungal agents for plants. However, no biological activity studies were reported.

**Scheme 31:**
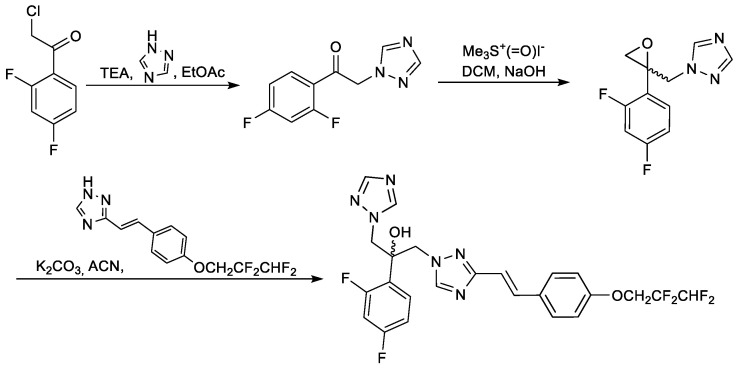
Synthesis of a novel fluconazole analog that had one of the 1,2,4-triazole rings substituted with a *(E)*-4-(2,2,3,3-tetrafluoropropoxy)styryl moiety at position C-3.

In 1988, F. Boyle et al. (Scheme 31, [43]) presented the synthesis of a new analog of fluconazole with modifications in the triazole ring. The objective was to develop a derivative with potentially enhanced antifungal activity, simultaneously mitigating adverse effects, especially those associated with teratogenicity.

In biological studies, the activity of the compound was compared with 24 other fluconazole analogs obtained similarly. Some of the compounds were obtained as single enantiomers by resolving as camphanic esters followed by hydrolysis. Attempts to find the structure–activity relationship were undertaken. In in vitro studies, IC_50_ parameters against *C. albicans* were determined, considering the spectrum of activity against eight different yeasts and eleven dermatophytes and the impact on human aromatase. In in vivo studies, the activity against vaginal candidiasis in mice was assessed, determining the lowest concentration that could lead to a complete cure. Additionally, the activity against aromatase in pregnant rats was examined by analyzing the placental mass after therapy. It was observed that the placental mass was higher by at least 30% than in the control group. The in vivo half-life of the compound was also determined by analyzing blood samples taken at 5, 24, 48, and 120 h post drug administration for 6 days. The compound demonstrated good antifungal activity both in vitro and in vivo: the IC_50_ against *C. albicans* was 0.003 µg/mL and the in vivo effectiveness reached 0.25 mg/kg.

**Scheme 32:**
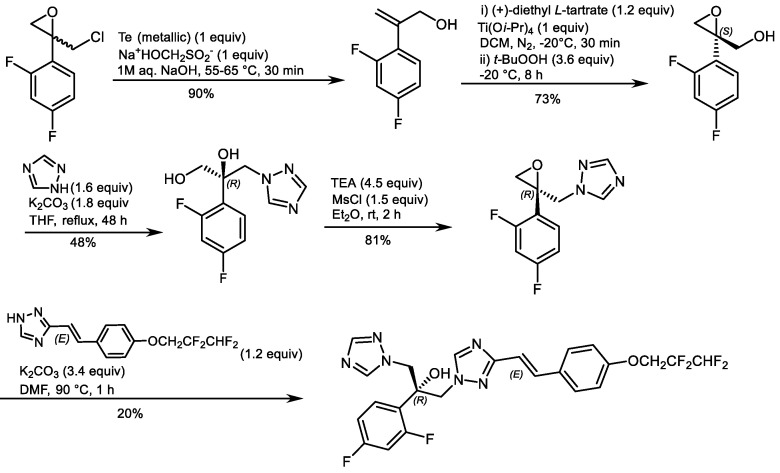
Synthesis of an enantiopure fluconazole derivative.

The same compound was patented four years later (1992) by Imperial Chemical Industries (Scheme 32, [44]) but as a single enantiomer achievement, which involved a substantially different approach. At the key steps, the presented approach involves (mediated by tellurium reagent formed in situ) transformation of racemic epichlorhydrine to an allylic alcohol followed by Sharpless asymmetric epoxidation. Obtaining enantiomerically pure epoxide allowed us to obtain enantiomerically pure analogs of fluconazole with no need for post-synthesis separation of enantiomers.

The parameters of in vitro activity (MIC) of the two optical isomers, compared to the activity of fluconazole, were determined against *C. albicans* IPM 40009, *Candida tropicalis* TIMM 0313, *Candida glabrata* TIMM 1064, *C. neoformans* T1AMM 0362, *T. mentagrophytes* TIMM 1188, and *A. fumigatus* 1FM 4942. The obtained compounds showed significantly higher activity than fluconazole, with the (+) isomer exhibiting MIC values toward the fungi in the range of 0.1–12.5 μg/mL. The MIC values of fluconazole were 25–100 μg/mL or higher. In vivo ED_50_ (effective dose) parameters for *C. albicans* IPM 40009, *C. neoformans* T1AMM 0362, and *A. fumigatus* 1FM 4942 were determined using a murine model and compared with those of fluconazole. The (+) isomer exhibited significantly higher activity, with values of 2.6, 19.7, and 39.8 mg/kg, respectively, compared to the values determined for fluconazole: >30, >100, and 57.9, respectively. The toxicity parameters were determined using a primate model, administering doses of 1, 3, 10, and 30 mg/kg/day. It was observed that the racemic mixture was safe at a dose of 1 mg/kg/day, while the (+) enantiomer exhibited toxic effects only at a dose of 30 mg/kg/day. The study demonstrated that the (+) enantiomer exhibited significantly stronger activity and lower toxicity than the (-) enantiomer and the racemic mixture [44].

**Scheme 33:**
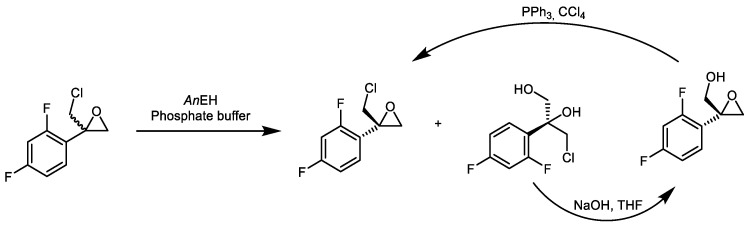
Enzymatic approach to the synthesis of an enantiopure key intermediate.

The enzymatic synthesis of the key intermediate of this fluconazole analog was also reported [45]. The authors used a fungal *A. niger* (*An*EH) epoxide hydrolase to resolve the racemic epoxide, as shown in Scheme 33. The process is based on presumably kinetically controlled enzymatic hydrolysis of *R*- enantiomer of epichlorhydrine to form chlorohydrin, which later was converted back to the epichlorhydrine, yet *S*- enantiomer, under the Appel reaction conditions.

**Scheme 34:**
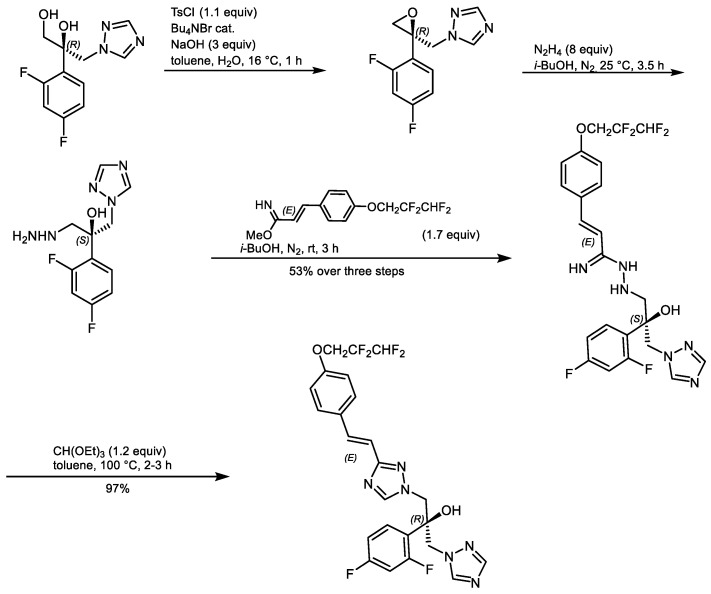
Alternative synthesis of an enantiopure fluconazole derivative with 2,2,3,3-tetrafluoropropoxystyryl substituted at the 1,2,4-triazole moiety.

In 1995, Zeneca Limited patented (Scheme 34, [46]) a new synthetic pathway for the analog of fluconazole mentioned above in Scheme 32.

**Scheme 35:**
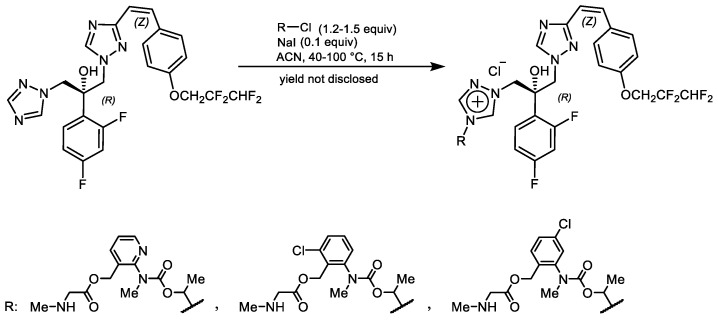
Synthesis of *N*-substituted carbamoyloxyalkyl-azolium analogs of fluconazole.

In 2001, F. Hoffmann-la-Roche (Scheme 35, [47]) patented the synthesis of three new *N*-substituted carbamoyloxyalkylazolium analogs of fluconazole with potential antifungal activity. Indeed, the approach was based on a post-synthesis modification of the already reported chiral non-racemic 2,2,3,3-tetrafluoropropoxystyryl analog, which involved functionalization of the nitrogen atom of the 1,2,4-triazole ring with biscarbamoyloxyalkyl groups. The obtained cationic analogs were not subjected to biological activity tests.

**Scheme 36:**
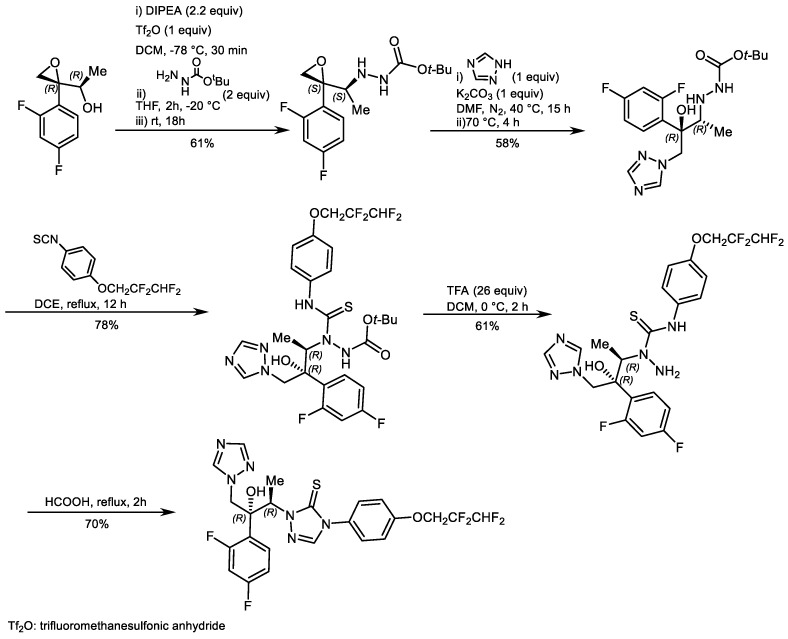
Synthesis of a new triazolethione analog of fluconazole.

In a patent from 2004, Ranbaxy Laboratories Limited (Scheme 36, [48]) claimed to have prepared a non-racemic triazolthione type of fluconazole analog that would be seen as a useful antifungal agent for treatment of animals and humans. The strategy was based on the initial condensation of *(1R*,*2R)*-1-[2-(2,4-difluorophenyl)oxiran-2-yl]ethanol with Boc-monoprotected hydrazine, followed by epoxide ring opening upon treatment with 1,2,4-triazole and three steps towards 2*H*-1,2,4-triazole-3(4*H*)-thione ring closing by the usual sequence of reactions. Notably, the structure of this analog was significantly different from that of the already well-established fluconazole type motifs: the well-documented 4-(2,2,3,3-tetrafluoropropoxy)styryl group was replaced by the simpler 4-(2,2,3,3-tetrafluoropropoxy)phenyl one, and the compound did not possess a second 1,2,4-triazol-1-yl unit; nevertheless, no biologic studies were reported.

**Scheme 37:**
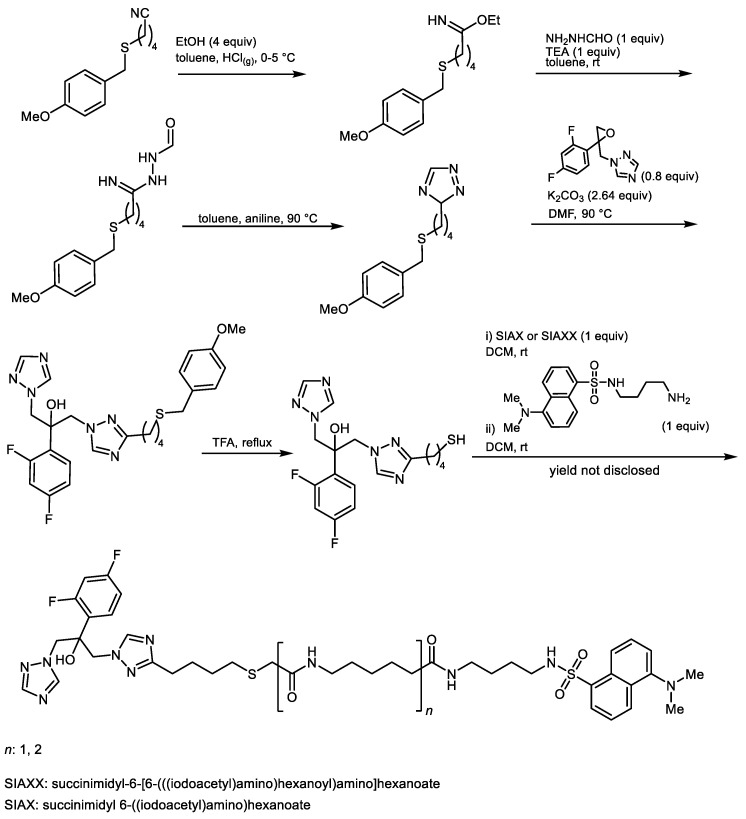
Synthesis of a dansyl–fluconazole conjugate.

**Scheme 38:**
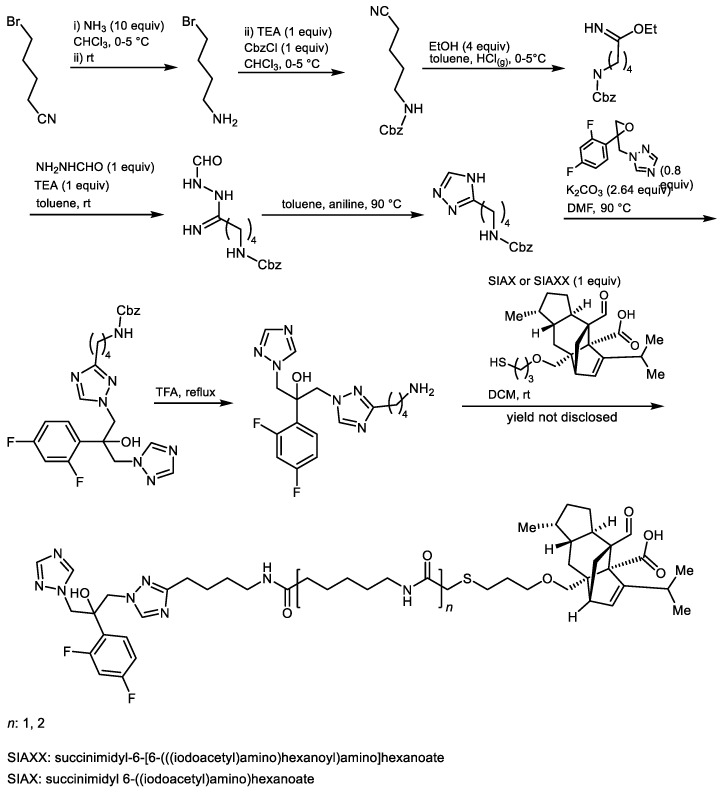
Synthesis of a sordarin–fluconazole conjugate.

In their patent from the year 2001, Brenner Sydney and coworkers described the synthesis of fluconazole conjugates comprising a vector-linker pharmacophore. They proposed introducing 4-aminobutyl and 4-sulfanylbutyl groups into the fluconazole structure to be used in the syntheses of conjugates. Two new analogs of fluconazole linked to the 5-(dimethylamino)naphthalene-1-sulfonyl (dansyl) group (Scheme 37) or the sordarin [49] molecule (Scheme 38) serving as a vector were obtained with that approach [50]. Sordarin was selected due to its affinity to the second molecular target located in the vicinity of the fluconazole target and dansyl as a fluorescent marker. Various linkers were employed in the synthesis of the compounds. However, no investigations regarding their biological activity were reported.

**Scheme 39:**
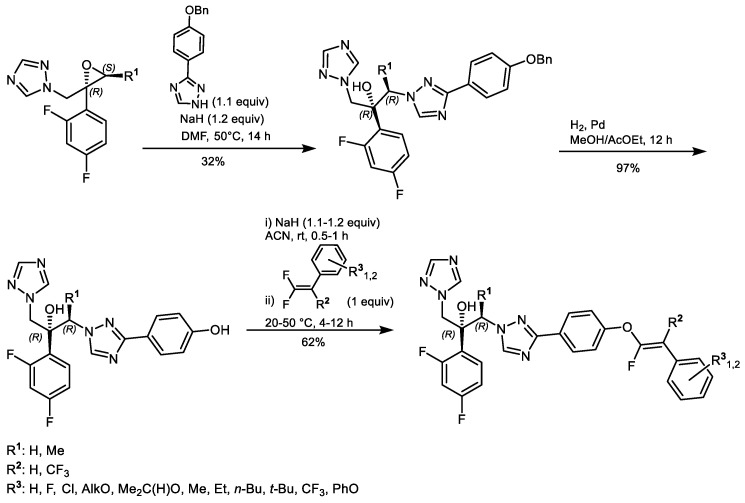
Synthesis of new fluconazole derivatives substituted at the triazole moiety.

In 2005, the Korea Research Institute of Chemical Technology issued a patent describing the synthesis of a medium library of antifungal analogs of fluconazole, featuring modifications in the 1,2,4-triazole ring (Scheme 39, [51]). The syntheses were based on the usual approaches applied to fluconazole, including oxirane ring opening with substituted triazoles. Some of the reported compounds also possessed substituents at the methylene moiety. To assess the in vivo antifungal capabilities, a mouse model was employed. Mouse survival was used as a parameter, with fluconazole as a positive control and a drug-free carrier as a negative control. The investigated compound, containing the 4-(((*Z*)-2-(4-chlorophenyl)-1-fluorovinyl)oxy)phenyl moiety (Scheme 38, R^1^: CH_3_, R^2^: H, R^3^: 4-Cl) at position 3 of the triazole ring, demonstrated lower antifungal efficacy than fluconazole but showed higher effectiveness than the drug-free carrier. Additionally, an analysis of hepatotoxicity parameters was conducted for the three described compounds, determining IC_50_ values for seven human cytochrome P450 isoenzymes in liver microsomes (CYP: 1A2, 2A6, 2C8, 2C9, 2C19, 2D6, 3A4). The oral toxicity of the compound was investigated using a mouse model, administering doses in the range of 62–2000 mg/kg body weight. The LD_50_ dose was determined to be 1750 mg/kg.

**Scheme 40:**
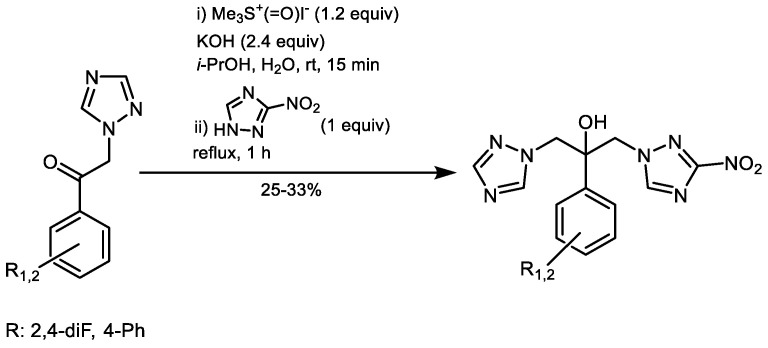
Synthesis of fluconazole analogs substituted at position 3 of the triazole moiety by a nitro group.

In 2015, M. V. Papadopoulou et al. (Scheme 40, [52]) published the results of the synthesis of 3-nitrotriazole derivatives. The synthesis of two new derivatives was described. Biological tests to determine their antiparasitic properties against factors causing neglected tropical diseases (NTDs), namely parasitic protists *Trypanosoma cruzi*, *Trypanosoma brucei rhodesiense*, and *Trypanosoma brucei gambiense*, were conducted. The compounds described in this study can inhibit the activity of the 14α-demethylase enzyme (CYP51) and function as prodrugs due to the activity of oxygen-insensitive type I nitroreductase (NTR), an enzyme that is absent in most eukaryotes. In vitro studies of activity against *T. cruzi* and *T. brucei rhodesiense* were performed, comparing the compounds to fluconazole and benznidazole. The compounds were also tested for toxicity against the rat skeletal (L6) myoblast cell line. Based on these studies, the selective index (SI = IC_50_ L6/IC_50_ parasite) was determined. The compound substituted with phenyl in position 4 of the carboaromatic ring (Scheme 40) exhibited significantly higher in vitro activity against *T. cruzi* than fluconazole and benznidazole (MIC = 0.033 μg/mL) and good selectivity (SI = 3807.7). However, it showed satisfactory activity against *T. brucei rhodesiense*, with an MIC of 2.887 μg/mL, and low selectivity (SI = 44). The in vitro activity of parasite enzymes TbNTR and TcNTR against these compounds was determined for their activation. The CYP51 inhibition ability of the compounds was assessed, and high inhibition ability of the compound substituted with phenyl and relatively low activity of the difluoro-substituted compound were shown. In vivo activity of these compounds was determined in a mouse model, demonstrating antiparasitic effects, i.e., a reduction in the parasitic index (PI) to >4% at doses of 15 mg/kg/day after 10 days. In vitro ADME parameters were determined for the compound substituted with phenyl, including Caco-2 permeability and metabolic stability in the presence of mouse and human microsomes. The compound exhibited high stability and permeability.

**Scheme 41:**
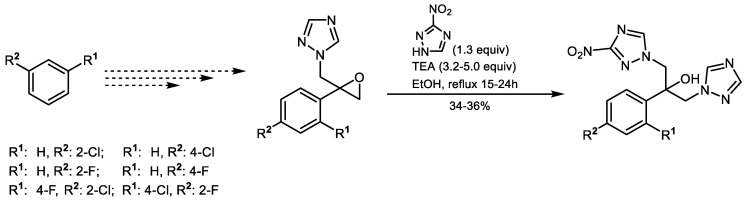
Synthesis of fluconazole derivatives substituted at the 3-position of the triazole ring with a nitro group.

In 2017, H. Sadeghpour et al. (Scheme 41, [53]) presented the results of their studies on the synthesis and bioactivity of new fluconazole derivatives with a nitro group positioned at the triazole moiety. The synthesis was accomplished according to the usual procedure covered by the first patent with minimal adaptations to the given substrates. The study included biological activity tests conducted in vitro against *Candida* and filamentous fungal strains, as well as computer simulations based on molecular docking. Most of the synthesized molecules exhibited satisfactory antifungal activity against filamentous fungi in vitro. This was associated with a study stating that derivatives of fluconazole with a 2,4-dichlorophenyl or 2,4-difluorophenyl group in the structure demonstrated higher biological potential than others. The dichloro-substituted compound showed the highest activity in the molecular docking tests and the lowest Final Docked Energy (FDE) for the 14α-demethylase of lanosterol. This compound also exhibited the highest antifungal activity among the tested compounds against *Microsporum gypseum*, with an MIC value of 4 µg/mL (for fluconazole: MIC = 8 µg/mL), and high antifungal activity against most *Candida* strains used in the tests, especially the clinical strain of *C. albicans*, with an MIC value of 0.5 µg/mL (for fluconazole: MIC = 1 µg/mL).

**Scheme 42:**
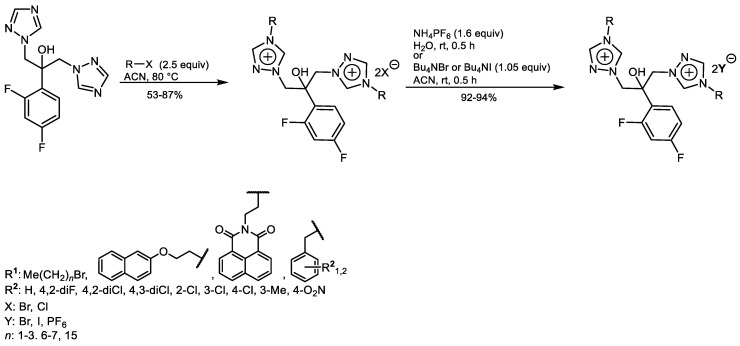
Synthesis of dicationic fluconazole analogs.

In 2011, Y-Y. Zhang et al. (Scheme 42, [54] described the synthesis of new analogs of fluconazole bearing additional substituents at both triazole moieties introduced by direct and regioselective *N*-alkylation carried out with substituted benzyl bromides, *n*-alkyl bromides, 2-(2-bromoethoxy)naphthalene, and *N*-(2-bromoethyl)-1,8-naphthalimide. Several non-bromide anions were also employed as counterions with fluconazoliums to evaluate their significance. MIC parameters were determined for Gram-positive bacteria (*Staphylococcus aureus* ATCC 6538, Methicillin-resistant *S. aureus* N315 (MRSA), and *Bacillus subtilis* ATCC 21216), Gram-negative bacteria (*Escherichia coli* ATCC 8099, *Pseudomonas aeruginosa* ATCC 27853, and *Proteus hauseri* ATCC 13315), and fungi (*C. albicans* ATCC 76615 and *A. fumigatus* ATCC 96918). Fluconazole, chloromycin, and norfloxacin were used as the positive controls. It is worth noting that the compounds synthesized in the study exhibited a broader antibacterial spectrum than fluconazole [52]; they were active against *S. aureus*, *P. hauseri*, *E. coli*, and *A. fumigatus* with an MIC in the range of 0.25–16 μg/mL.

**Scheme 43:**
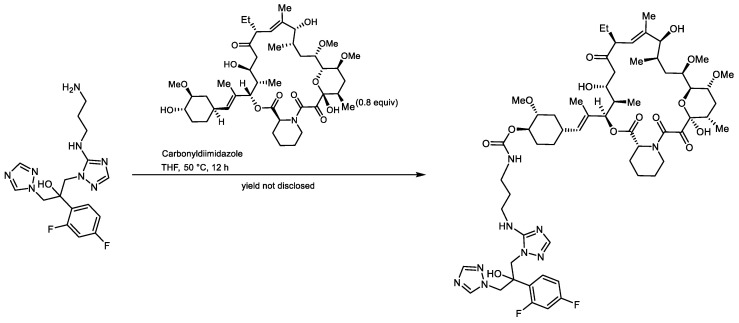
Synthesis of a conjugate of fluconazole with ascomycine.

In 2012, Amplyx Pharmaceuticals Inc. patented the synthesis of a conjugate of fluconazole with ascomycine, a bioactive compound with antifungal and immunosuppressive properties, through linkers covalently bonded to the triazole ring (Scheme 43, [55]). Initially, a fluconazole derivative possessing amino and isocyanate groups was obtained. The connection of two active molecules was realized by the formation of a carbamate moiety.

**Scheme 44:**
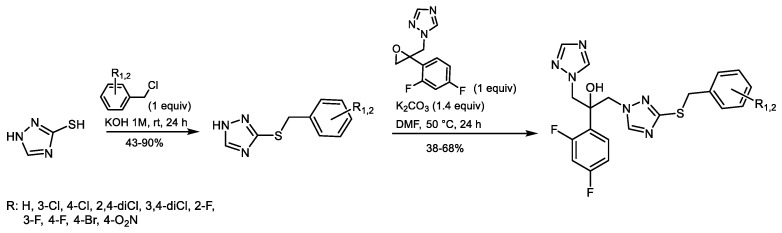
Synthesis of fluconazole analogs decorated with benzylthio groups.

K. Motahari et al. (Scheme 44, [56]) presented a series of fluconazole analogs with variously functionalized benzyl groups grafted at the C-3 position of 1,2,4-triazole. They conducted in vitro biological studies to determine MIC values against fluconazole-susceptible *Candida* species, including *C. albicans*, *C. glabrata*, *C. parapsilosis*, *C. krusei*, and *C. tropicalis*. All the compounds exhibited stronger activity against the nonalbicans *Candida* species than fluconazole, especially against *C. parapsilosis*. The derivative with the 2,4-dichloro benzylthio substituent showed the highest activity against *C. albicans*, with an MIC value of 0.125 μg/mL, compared to fluconazole with an MIC of 1 μg/mL. In turn, the 3-chlorobenzylthio analog (R: 3-Cl, Scheme 43) was the most active compound against the nonalbicans *Candida* species, with MIC values ranging from 0.063 to 0.125 μg/mL, in comparison to fluconazole with MICs in a range from 0.5 to 4 μg/mL. The compound exhibited a broad profile of activity against fluconazole-resistant *Candidia* strains, such as *C. albicans* (IFRC 1260), *C. albicans* (IFRC 1261), *C. albicans* (IFRC 1262), *C. parapsilosis* (IFRC 84), *C. krusei* (IFRC 1280), and *C. krusei* (IFRC 1281. The two other derivatives of the series (R: 4-Br and 3,4-diCl) were characterized by better activity towards these species of fungi. The cytotoxicity studies against human hepatoma cells Hep-G2 and the normal cell line NIH-3T3 revealed the IC_50_ value ≥ 83.2 μg/mL, while the value for fluconazole was ≥199.3 μg/mL.

**Scheme 45:**
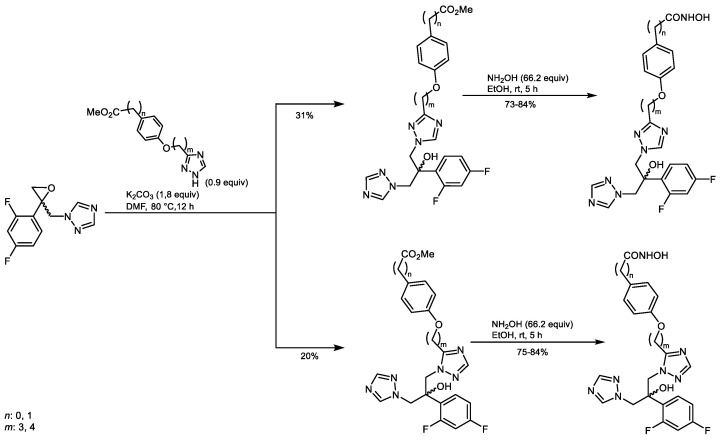
Synthesis of fluconazole-based CYP51/HDAC dual inhibitors.

In 2020, G. Han et al. (Scheme 45, [57]) reported the synthesis of fluconazole conjugates with compounds acting as histone deacetylase inhibitors to enhance their antifungal efficacy against fluconazole-resistant strains. The synthesized CYP51/HDAC dual inhibitors were subjected to antifungal activity assessments against azole-sensitive *C. albicans* and *C. neoformans* as well as four azole-resistant clinical isolates of *C. albicans* and *C. tropicalis*. The MIC_80_ parameter (minimum inhibitory concentration with inhibition over 80%) was determined and compared to that of fluconazole, itraconazole, and voriconazole. Some of the compounds exhibited increased activity against the azole-resistant *C. albicans* strains, with MIC_80_ ranging from 8 to 64 μg/mL, compared to fluconazole, itraconazole, and voriconazole, which had MIC_80_ values exceeding 64 μg/mL. However, the activity against the azole-sensitive strains was significantly lower than that of the reference drugs, with MIC_80_ ranging from 4 to 64 μg/mL, and voriconazole achieving an MIC_80_ of 0.125 μg/mL.

**Scheme 46:**
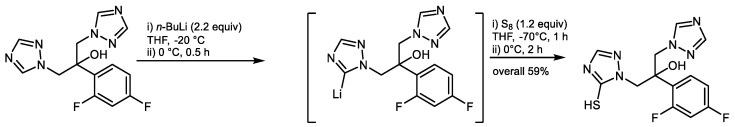
Synthesis of a 3-mercapto-1,2,4-triazole analog of fluconazole.

In two patents from 1997, Bayer AG [58,59] disclosed the synthesis of new sulfur-containing fluconazole analogs and proposed their applications as microbicides for plant protection and preservation of materials. The authors conducted their synthesis via direct lithiation and sulfuration of the organolithium intermediate by molecular sulfur as an eletrophile (Scheme 46). Details of biological studies of the obtained derivatives were not reported.

## 4. Derivatization of the Hydroxy Group

### 4.1. Esters

**Scheme 47:**
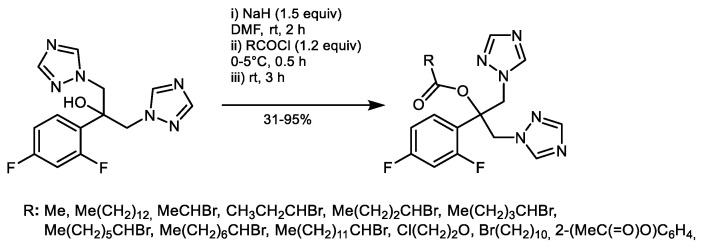
Synthesis of new fluconazole esters obtained from selected acid chlorides.

**Scheme 48:**
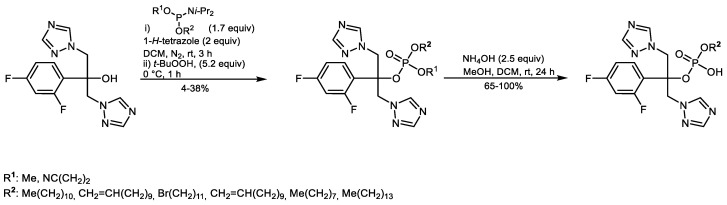
Synthesis of phosphate derivatives of fluconazole.

Keykavous Parang et al. described the synthesis and biological investigations of two classes of fluconazole derivatives: carboxylic acid esters and carbohydrate phosphate esters (Scheme 47 and Scheme 48) [60,61].

The antifungal properties of carboxylic acid esters against *C. albicans* ATCC 14053, *C. neoformans* ATCC 66031, and *A. niger* ATCC 16404 were determined in SBD (Sabouraud Dextrose Broth) medium. For *C. albicans*, an additional test involving RPMI medium was conducted. All the derivatives showed weaker activity than fluconazole in RPMI (Roswell Park Memorial Institute medium); however, in the SBD medium, they were active against *C. albicans* while fluconazole was inactive. It was observed that these derivatives were more lipophilic, potentially contributing to better skin penetration. The derivatives substituted with bromine were found to exhibit higher chemical stability. Phosphate di- and triesters with fluconazole and fatty alcohols were investigated. Some of them showed stronger antifungal activity against *C. albicans*, *C. neoformans*, and *A. niger* than fluconazole. Notably, two compounds—*O*-2-bromooctanoylfluconazole and *O*-11-bromoundecanoylfluconazole—were particularly active against *C. neoformans*. The MIC values (defined as the lowest concentration of the drug that inhibits the visible growth of microorganisms after 48 h of incubation) were 111 µg/mL and 198 µg/mL, respectively, whereas fluconazole had MIC ≥ 4444 µg/mL. Among all the phosphates (Scheme 47), the highest activity was determined in the case of 2-cyanoethyl-ω-undecanyl fluconazole phosphate (MIC = 122 µg/mL against *C. albicans*, fluconazole MIC > 4444 µg/mL) and methyl-undecanyl fluconazole phosphate (MIC = 190 µg/mL against *A. niger*, fluconazole MIC > 580 µg/mL).

**Scheme 49:**
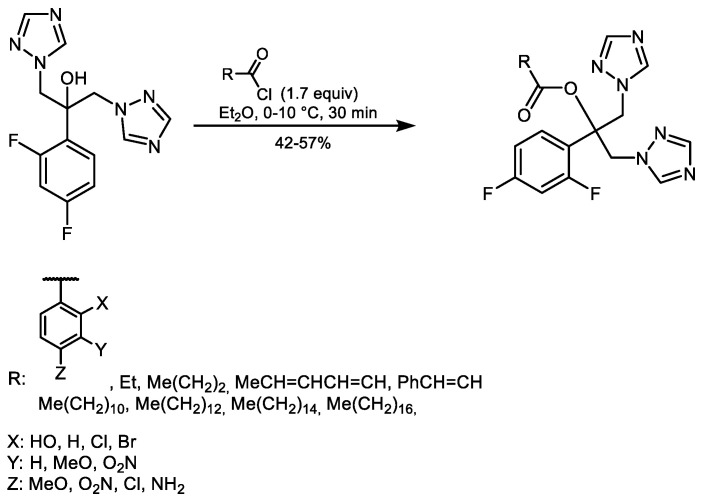
Synthesis of new fluconazole derivatives using acid chlorides.

In 2007, the synthesis of esters with fluconazole using acid chlorides of various aliphatic and aromatic carboxylic acids was described (Scheme 49, [62]). In vitro activity tests were performed against *C. albicans* and *A. niger*. Fluconazole myristate, veratrate, and stearate exhibited the strongest activity, achieving pMIC values (µM/mL) of 2.54, 2.65, and 2.76, and 2.84, 2.85, and 2.96 against *A. niger*, respectively. All these compounds were more active than fluconazole. Additionally, a QSAR analysis was carried out, demonstrating that the use of the energy of the lowest unoccupied molecular orbital (LUMO) and the second-order molecular connectivity index effectively allows the prediction of the antifungal capabilities of new fluconazole derivatives.

**Scheme 50:**
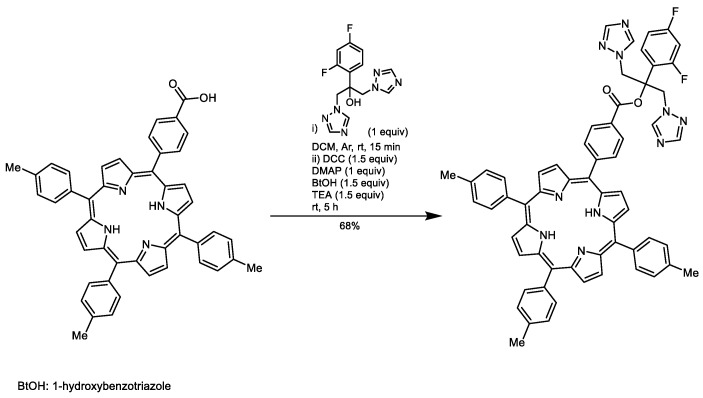
Synthesis of a fluconazole–porphyrin conjugate.

The novel derivative of fluconazole that combines fluconazole with porphyrin (Scheme 50) was synthesized by Mora et al. in 2010 [63]. The aim of their work was to obtain a photosensitive compound that could be used in the photodynamic inactivation (PDI) of microorganisms. In this process, a combination of light and a photosensitizer was employed. The study involved measurements of the photodynamic activity in various media with comparison to a fluconazole-free homolog, porphyrin-methyl ester (P-est) and determination of the photoinactivation efficiency against *C. albicans* in a cellular suspension.

The compound exhibited lipophilic properties resulting in low water solubility. Thus, beta-cyclodextrins were used as vehicles. It was demonstrated that the compound synthesized in this study was sensitive to light, and its degradation occurred both in dimethylformamide (DMF) and water. The compound was shown to inhibit the growth of *C. albicans* both in the dark and with visible-light exposure, with higher activity observed under the light exposure. However, this activity was comparable to that observed when fluconazole was administered with non-covalently linked P-est and higher than that of P-est without added fluconazole.

**Scheme 51:**
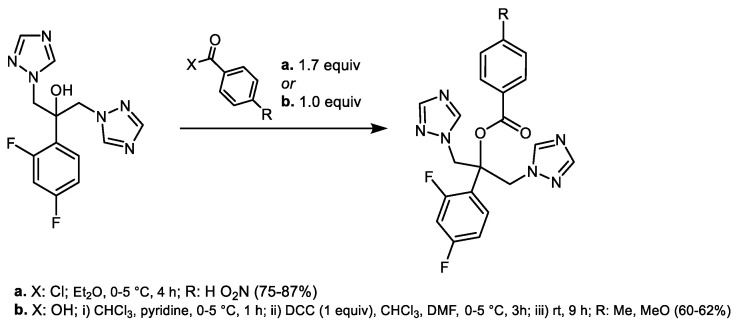
Synthetic routes to fluconazole benzoic acid esters.

In 2010, scientists from India synthesized and evaluated fluconazole esters derived from appropriate 4-substituted benzoic acids as potential antifungal agents [64]. The compounds were obtained through the reaction of fluconazole with the corresponding acid chloride or directly through condensation with the appropriate carboxylic acid in the presence of *N*,*N*-dicyclohexyl carbodiimide (DCC) (Scheme 51).

MIC values were determined using the broth dilution method. The *C. albicans* ATCC 10231 strain was used in the research. The results were compared with those for two drugs: fluconazole and ketoconazole. Additionally, the MEP (Minimal Effective Concentration) was determined. The MEP is the lowest concentration of a drug at which the production of fungal metabolites is inhibited by at least 50%. The MEP results corresponded to the outcomes of the MIC tests. Fluconazole *p*-metoxybenzoate showed stronger activity than fluconazole, achieving MIC = 1.5 μg/mL, while fluconazole benzoate exhibited activity comparable to that of fluconazole, with MIC = 2 μg/mL.

**Scheme 52:**
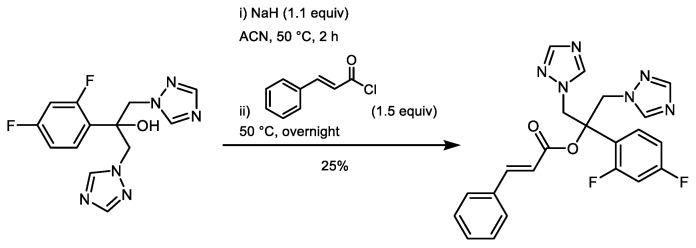
Reaction between fluconazole and cinnamic acid chloride.

The synthesis of esters of cinnamic acid with various antifungal drugs, including fluconazole, was described by De Vita et al. in 2016 (Scheme 52, [65]). The reaction was performed using cinnamic acid chloride in the presence of sodium hydride in acetonitrile. Subsequently, tests were conducted to determine the ability of the obtained compounds to inhibit *C. albicans* ATCC 10231 biofilm formation. The derivative of fluconazole exhibited increased inhibitory activity against biofilm formation, with a BMIC_50_ value (≥50% biofilm inhibition concentrations) of 4 μg/mL, compared to cinnamic acid with BMIC_50_ > 128 μg/mL.

The poor solubility of fluconazole prompted the search for water-soluble prodrugs of fluconazole, and this search has been ongoing since the mid-1990s [66]. They were obtained, among others, by chemical modification within the hydroxyl group of fluconazole. The modification resulted in a significant improvement in pharmacokinetic properties. However, there are no data on the impact of this type of modification on antifungal activity.

**Scheme 53:**
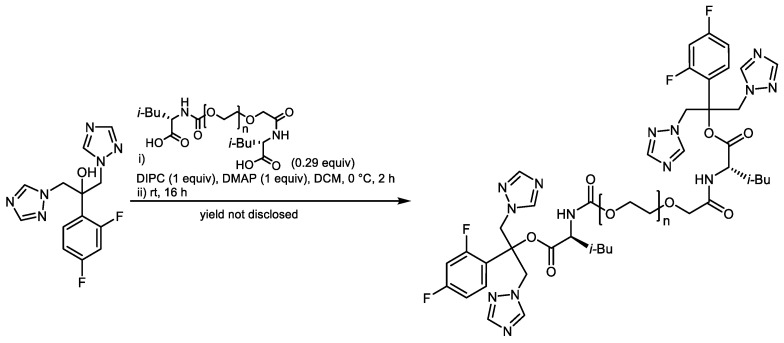
Synthesis of two fluconazole molecules linked by PEG.

Enzon Pharmaceuticals proposed a method for the synthesis of a derivative of fluconazole by connecting two fluconazole units through 40 kDa polyethylene glycol Leucine diacid (Scheme 53). This modification was applied to improve pharmacokinetic parameters and reduce the toxicity of the drug, with a specific focus on optimizing the rate of metabolism and, consequently, the release of the active form of the drug. No biological tests were reported [67].

**Scheme 54:**
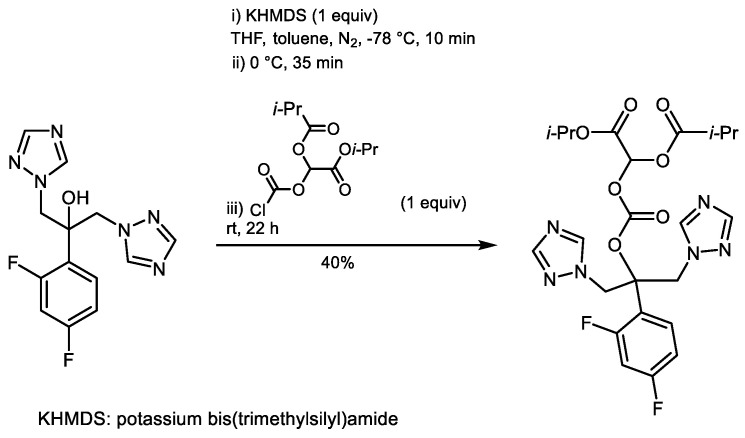
Synthesis of 2-methylpropionic acid [(2,4-difluoro-1-phenyl)-bis-(1*H*-1,2,4-triazol-1-ylmethyl)]methyloxycarbonyloxy-(2-propyloxycarbonyl)methyl ester.

Rohm and Haas Company patented a method of synthesizing a new ester of fluconazole in the reaction with 1-[(chlorocarbonyl)oxy]-2-oxo-2-[(propan-2-yl)oxy]ethyl 2-methylpropanoate (Scheme 54). Neither the option for such a sophisticated esterification partner nor bioactivity tests of the resulting ester were reported in the patent [68].

However, three years later, the same company patented the structure of new combinations involving many commonly used drugs, including fluconazole, determined with the previously described procedure. The study aimed to obtain compounds with improved pharmacokinetic parameters compared to the existing drugs. All the new compounds were designed to have enhanced permeability through biological membranes [69].

**Scheme 55:**
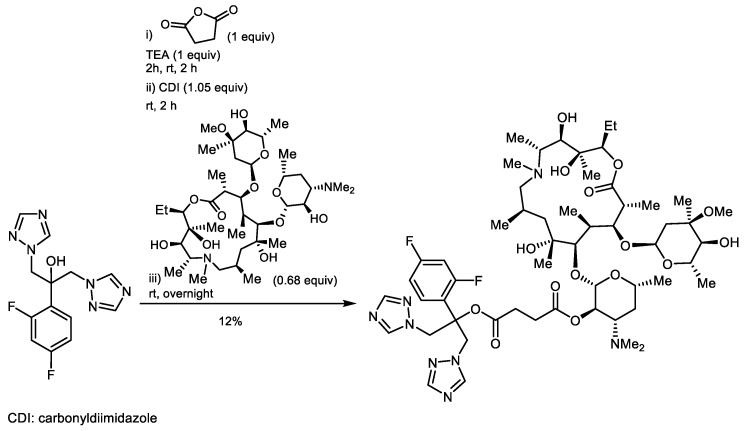
Synthesis of a new fluconazole derivative with an azithromycin moiety.

In 2003, scientists from Sympore GmbH patented the results of similar studies aimed at enhancing pharmacokinetic properties. They described a two-step synthesis of a succinic diester connecting (i) fluconazole and (ii) the macrolide azithromycin (Scheme 55, [70]).

**Scheme 56:**
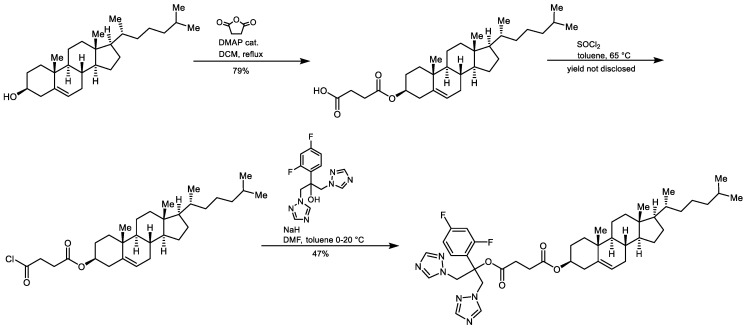
Synthesis of cholesterol-fluconazole conjugate.

In 2010, SEPS Pharma N.V. patented the structure of a new derivative of fluconazole, a diester of succinic acid with fluconazole and cholesterol (Scheme 56) available in a three-step synthesis [71]. In contrast to the aforementioned procedure, after *O*-acylation of cholesterol with succinic anhydride, the resulting carboxyl monoester required preliminary conversion into acid chloride before the second *O*-acylation, i.e., that of fluconazole. The solubility of the product in solvents used in pharmacy (water, water mixed with vitamin or polymers) was determined. The highest solubility was determined in the solvent H_2_O/vitamin E TPGS NP (90/10)—1.7 mg/mL. Chemical stability was also investigated in H_2_O (pH = 7), H_2_O (pH = 10), H_2_O/hydroxypropyl-β-cyclodextrin (60/40), and polyethylene glycol 400 by determining the percentage of the remaining compound using LCMS after 4, 24, 48, 168, and 336 h. After this time, the concentration of the compound decreased by more than 1% only in H_2_O pH = 10.

**Scheme 57:**
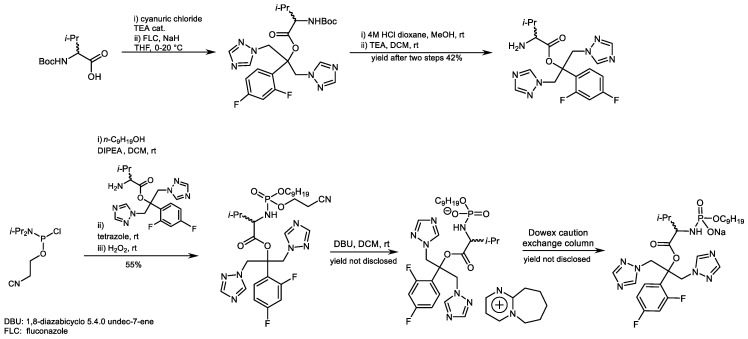
Synthesis of sodium nonyl {1-{[2-(2,4-difluorophenyl)-1,3-di(1*H*-1,2,4-triazol-1-yl)propan-2-yl]oxy}-3-methyl-1-oxobutan-2-yl}phosphoramidate.

The same company described a multistep synthesis of new fluconazole esters containing *N*-phosphorylated DL-valine moiety (Scheme 57, [72]). The application of this type of modification allows the formation of new formulations, such as microemulsion, nanoemulsion, and micro-suspension, resulting in novel bioavailability. Another purpose of this formulation was to extend the drug’s duration of action in the body and limit the number of doses administered throughout the day. Simultaneously, the presence of the phosphate group ensures better solubility of the drug in water. The solubility was determined in solvents commonly used in pharmacy, such as water and water with cyclodextrin, representing solubility above 10 mg/mL. Additionally, the chemical stability of the compound was evaluated in H_2_O (pH = 7), H_2_O (pH = 10), and H_2_O/hydroxypropyl-β-cyclodextrin (60/40) by determining the percentage of the remaining compound using LCMS after 2, 4, 24, and 168 h.

**Scheme 58:**
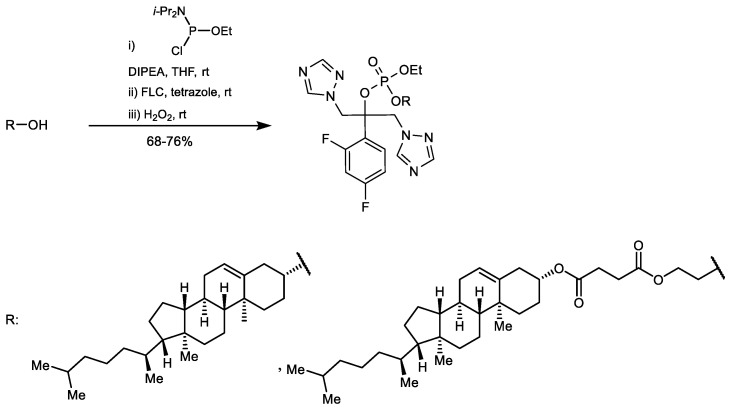
Synthesis of a fluconazole hybrid with cholesterol linked with a phosphate group.

**Scheme 59:**
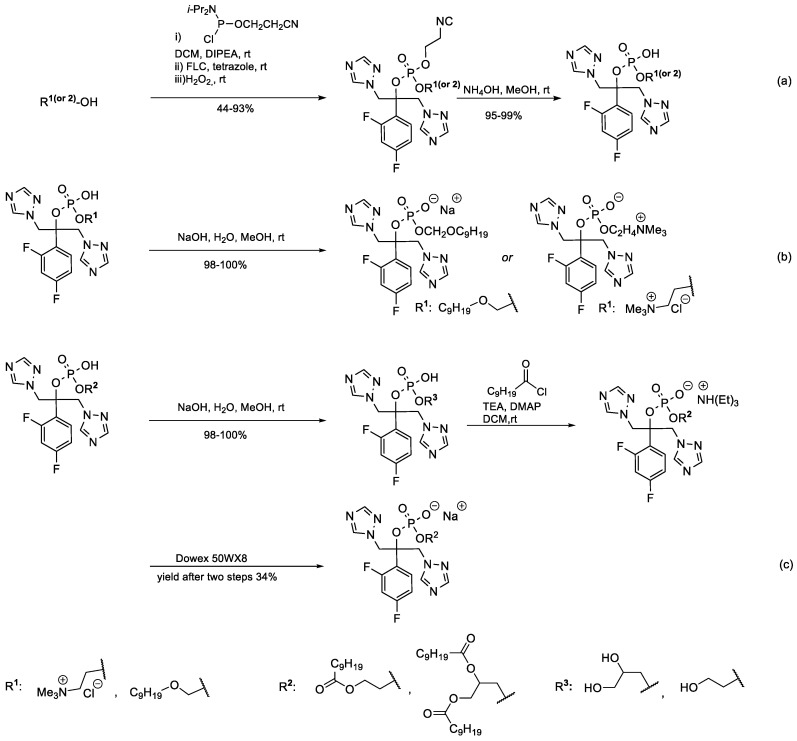
Synthesis of fluconazole derivatives with different alcohols linked with a phosphate group: with substituents R^1^ and R^2^ (**a**), with substituents R^1^ (**b**), with substituents R^2^ (**c**).

In a similar context and in search of compounds with improved pharmacokinetic parameters, two years later, Gonnissen et al. from Seps Pharma N.V. patented the synthesis of fluconazole derivatives, which were phosphate diesters (Scheme 58 and Scheme 59). The purpose of this work was to obtain a prodrug released over an extended period in the body upon intravenous administration, thereby reducing the number of administered doses while maintaining high solubility and, consequently, a low injection volume. The solubility and stability of the obtained compounds in various pharmaceutical solvents were determined, showing both good chemical stability and solubility for some of the compounds, mostly in non-polar solvents [73].

**Scheme 60:**
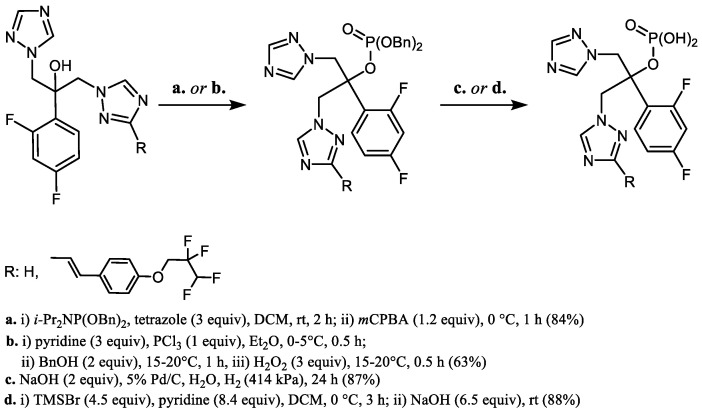
Synthesis of fluconazole phosphate.

New phosphorylated derivatives of fluconazole were described by Murtiashaw et al. (Scheme 60, [74]) in the patent of Pfizer Research and Development Co. The compounds were obtained via reactions with dibenzyl dipropan-2-ylphosphoramidoite or using PCl_3_ in combination with benzyl alcohol as an alternative approach. Subsequently, the resulting compounds underwent a reduction on palladium to obtain monoesters. The solubility properties of the derivative of fluconazole with a phosphate group were compared to that of fluconazole, revealing significantly greater solubility for this derivative. The same processes were generalized and reported again in 2002 [66].

### 4.2. Ethers

**Scheme 61:**
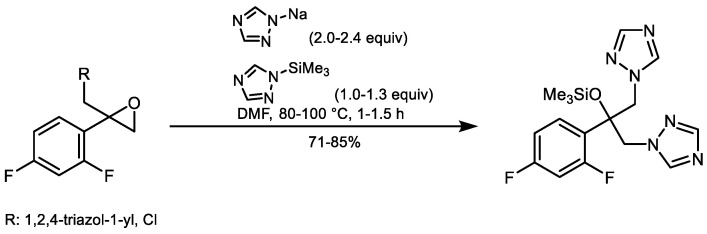
Synthesis of *O*-TMS protected fluconazole.

In 1994, Richter Gedeon Vegyeszeti Gyar Rt. (Scheme 61, [75]) proposed a two-step synthesis of an *O*-TMS derivative of fluconazole. A study of antifungal activity was conducted by determining the MIC parameter in vitro against *C. albicans*, comparing the compound to fluconazole. The derivative exhibited significantly higher activity than fluconazole with an MIC value of 150 μg/mL, compared to the MIC of 2500 μg/mL of fluconazole.

**Scheme 62:**
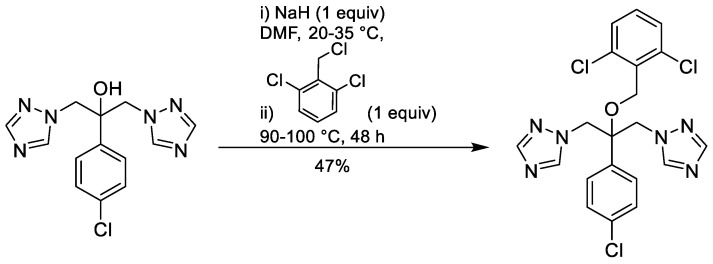
Synthesis of a fluconazole analog as ether derivative.

As shown earlier [28], in the same patent of Imperial Chemical Industries PLC, the synthesis of 2,6-dichlorobenzyl ether of a fluconazole analog was achieved in typical Williamson reaction conditions (Scheme 62, [28]).

### 4.3. Halo Analogs of Fluconazole at the Carbinolic Center

**Scheme 63:**
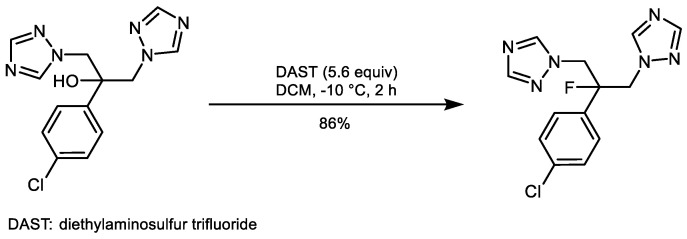
Synthesis of an analog of fluconazole with fluorine atom instead of hydroxy group.

In 1984, Schering Corporation patented a method of the synthesis of a fluconazole derivative in which a fluorine atom has replaced the hydroxy group, with the aromatic ring bearing one chlorine atom in position 4 instead of two fluorine atoms (Scheme 63). Despite the potential of the new substance to be active against fungi, no biological tests were reported [76].

**Scheme 64:**
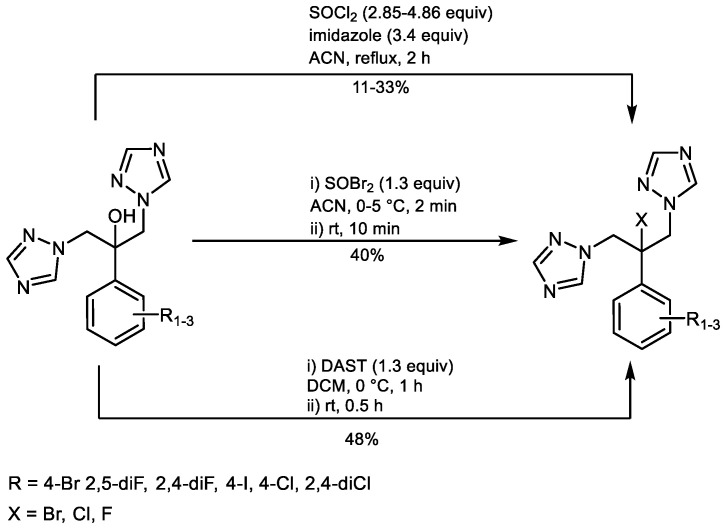
Synthesis of fluconazole derivatives without the hydroxy group.

Narayanaswami et al. (Scheme 64, [77]) patented the synthesis of new fluconazole derivatives with a fluorine atom replacing the hydroxy group and modifications within the aromatic ring. These compounds may have applications in treating fungal infections in both animals and humans as well as potential applications in agriculture. The PD_50_ parameter of activity of these compounds upon oral administration in mice ranged from 0.1 to 1.6 mg/kg, which suggests that these derivatives exhibited significant activity at relatively low doses in preclinical studies.

**Scheme 65:**
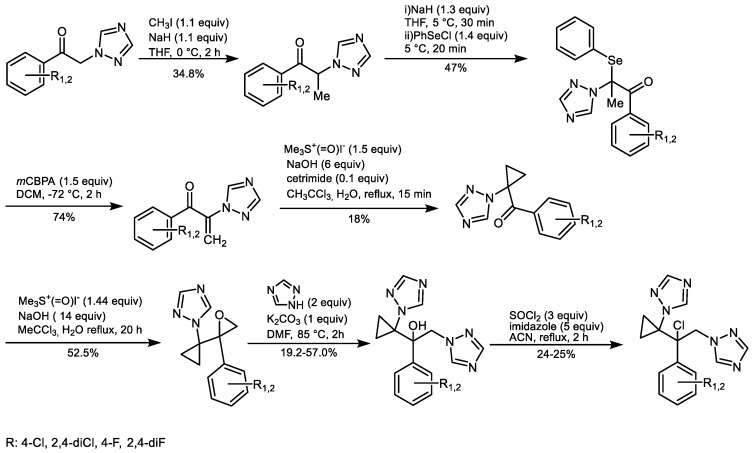
Synthesis of chloro analogs of fluconazole.

Pfizer Ltd. developed synthetic routes by which to obtain chloro analogs of fluconazole which consisted of replacing one of the methylene connections by a cyclopropane-1,1-diyl group and various substitutions in the benzene ring (Scheme 65). The PD_50_ parameter (50% protection against the lethal effect of the injection) was determined in vivo. The PD_50_ was assessed after 48 days. All the compounds exhibited activity, with the most active 1-{2-chloro-2-(4-fluorophenyl)-2-[1-(1*H*-1,2,4-triazol-1-yl)cyclopropyl]ethyl}-1*H*-1,2,4-triazole having PD_50_ values of <1 mg/kg. The value of this parameter for fluconazole has not been determined [78].

### 4.4. Fluconazole Analogs Comprising Other Groups at the Carbinolic Center

**Scheme 66:**
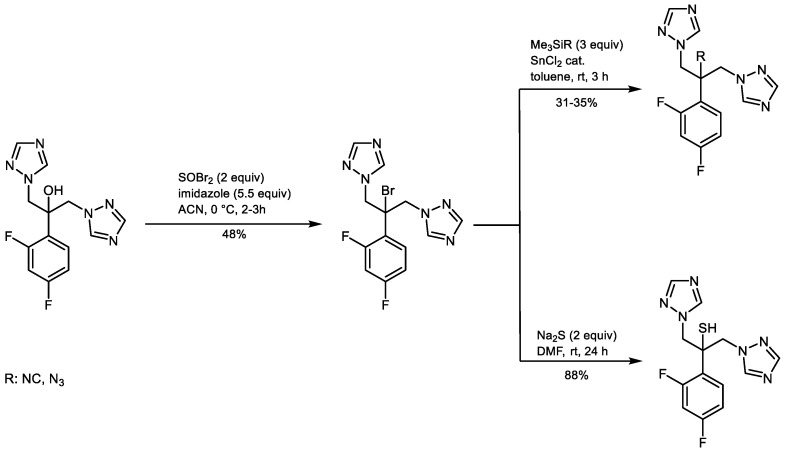
Synthesis of novel analogs (cyano, azido, and mercapto) of fluconazole at the carbinolic center.

As demonstrated earlier, Parang et al. [61] presented the synthesis of eight analogs (cyano, azido, and mercapto) of fluconazole at the carbinolic center (Scheme 66). Using HydroWin software (Version 1.67), it was determined that the compounds in SDB medium were chemically stable and did not undergo significant hydrolysis. The skin permeability parameter of all the newly synthesized derivatives was higher than that of fluconazole. Studies on the antifungal activity of derivatives substituted at the hydroxyl group were conducted against *C. albicans* ATCC 14053 in RPMI medium, *C. albicans* ATCC 14053 in SDB medium, *C. neoformans* ATCC 66031 in SDB medium, and *A. niger* ATCC 16404 in SDB medium. The compound with the SH group was two-fold more active towards *C. albicans* in SBD medium in comparison to fluconazole, and the compound with the chloro substituent was two-fold more active in RPMI medium [61].

A few earlier limited reviews were published in 1996 [79], 2002 [66], and 2006 [80].

## 5. Conclusions

The present work is an overview of the methods of the synthesis and biological activity of fluconazole itself, as well as its analogs and derivatives.

Several reported fundamental modifications have been made to the structure of fluconazole to enhance its bioactivity (selectivity and availability) along with the increase in solubility in water/lipids. These modifications involve alteration of the hydroxyl group, mainly through its esterification or etherification, replacement of the hydroxy group with other function, changes in the carboaromatic moiety, changes at the heteroaromatic moiety, introduction of additional substituents at methylene groups, and a combination of these modifications.

A greater potency is often achieved by replacing the hydroxyl group with another substituent, e.g., formation of ethers. There are only two literature reports dedicated to such compounds, but the obtained structures exhibited over ten-fold higher activity against *Candida* strains than fluconazole. However, good water solubility of fluconazole was achieved by phosphorylation of the hydroxyl to form the fluconazole mono ester of phosphorus acid.

Modifications involving changes in the fluorophenyl substituent also affect the potency and selectivity of the antifungal drug against specific fungal strains. Optimal modification can enhance effectiveness against fungal pathogens while simultaneously minimizing the impact on host cells. This, in turn, reduces the occurrence of side effects and increases the safety of the therapy. Alterations in the fluorophenyl group influence the pharmacokinetics of the drug, e.g., the solubility and absorption from the gastrointestinal tract, bioavailability, and distribution in the body. These factors are crucial for therapeutic effectiveness.

Modifications in the 1,2,4-triazole ring involve various chemical strategies, such as the introduction of substituents or the replacement of heterocyclic rings. These changes allow the synthesis of new derivatives of fluconazole that may exhibit increased antifungal activity while simultaneously minimizing potential side effects. They represent a significant step towards developing more effective and adaptable antifungal drugs in response to the evolving pathogen resistance. Understanding the impact of modifications in this segment may open new perspectives in the design of drugs aimed at more precise and efficient combating of fungal infections.

Commonly, in the presented studies, more than one modification to the structure was applied because diverse modifications to the structure of fluconazole aim to improve therapeutic properties and pharmacokinetics and at the same time prevent the development of resistance. This approach can lead to better clinical outcomes and the development of modern antifungal therapies.

Many new derivatives exhibit comparable or higher biological activity than fluconazole, especially when clinical strains of *C. albicans* are used. Moreover, some of them exhibit superior pharmacokinetic qualities and reduced toxicity in comparison to fluconazole. A comparison of the biological activity of the analogs and derivatives of fluconazole which were mentioned above can be found in the Supplementary Materials.

## Data Availability

Data are contained within the article and Supplementary Materials.

## Supplementary Materials

The following supporting information can be downloaded at: https://www.mdpi.com/article/10.3390/molecules29122855/s1. Comparison of biological activity of analogs and derivatives of fluconazole. Reference [81] is cited in Supplementary Materials.
