# Supplementary material for: Fluconazole Analogs and Derivatives: An Overview of Synthesis, Chemical Transformations, and Biological Activity
Source: Molecules. 2024 Jun 15;29(12):2855. doi: 10.3390/molecules29122855 (PMC11206329; doi:10.3390/molecules29122855)
Supplement: Supplementary file 1 [file molecules-29-02855-s001.zip › molecules-2964942-supplementary.pdf]

Supplementary Materials

Fluconazole Analogs and Derivatives: An Overview of Synthesis, Chemical Transformations, and Biological Activity

Michał Janowski [1], Oleg M. Demchuk [2*] and Monika Wujec [3*]

[1] Doctoral School, Medical University of Lublin, Chodzki 7, 20-093 Lublin, Poland
[2] Faculty of Medicine, The John Paul II Catholic University of Lublin, Konstantynow 1J, 20-708 Lublin, Poland; Oleh.Demchuk@KUL.Lublin.pl
[3] Department of Organic Chemistry, Faculty of Pharmacy, Medical University of Lublin, Chodzki 4a, 20-089 Lublin monika.wujec@umlub.pl
* Correspondence: Oleh.Demchuk@KUL.Lublin.pl

Comparison of biological activity of analogous and derivatives of fluconazole

Structure	Biological tests	Results
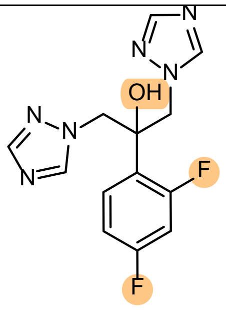<br>1982 [1]	• *In vitro* antifungal activity against 35 strains of *C. albicans.*<br><br>• *In vitro* antifungal activity in different pH<br><br>• *In vivo* survival of rats infected with *C. albicans*	• MIC in range of 0.063 - 4.0 μg/mL, with MIC of 0.25 μg/mL for 90% of strains<br><br>• Highest activity with pH of 7.4<br><br>• Survival rate of 94% at dose of 0.5 mg/kg per day [2]
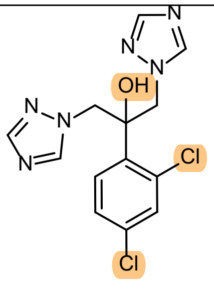<br>1982 [3]	• Inhibition of fungal infection in plants<br><br>• Inhibition of plant growth	• Significantly inhibited fungal infection<br><br>• Plant Growth was not significantly inhibited

Supplementary Materials

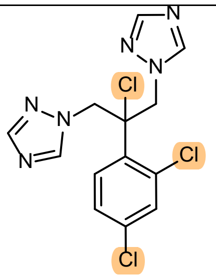 1983 [4]	• *In vivo* antifungal activity against *C. albicans* in mice	• PD_{50} 0.1 mg/kg
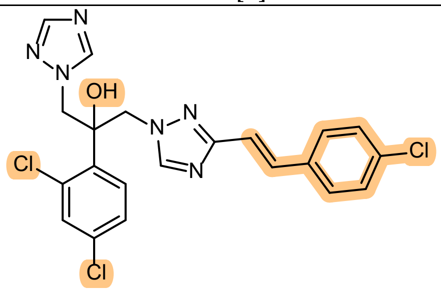 1984 [5]	• *In vitro* MED in mouse model infected with *C. albicans* and *T. mentagrophytes*	• MED 5 mg/kg
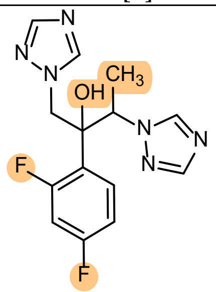 1984 [6]	• *In vivo* studies of PD50 and MST	• PD value was 0.1 mg/kg<br>• Extended MST by 20 days compared to ketoconazole
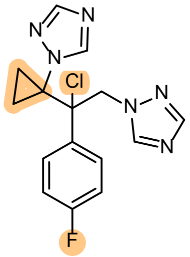 1985 [7]	• *In vivo* antifungal activity against *C. albicans* in mice	• $PD_{50} < 1$mg/kg
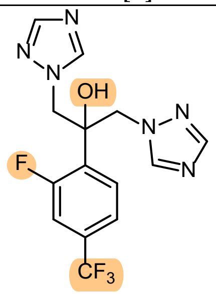 1985 [8]	• *In vivo* Minimum Effective Dose (MED) for *C. albicans* and *T. mentagrophytes* in rats and rabbits	•MED was determined to be 10 mg/kg, and for rats, it was 25 mg/kg.

Supplementary Materials

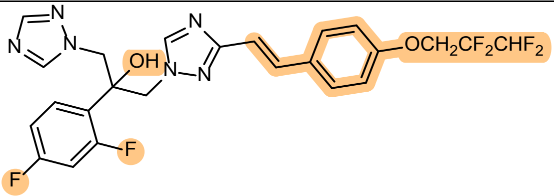<br>1988 [9]	• *In vitro* and *In vivo* activity against *C. albicans*	• IC50 0.003 µg/mL<br>• MED 0.25 mg/kg
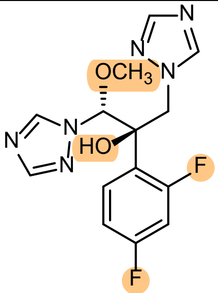<br>1989 [10]	• *In vitro* studies against *C. albicans, A. fumigatus, T. asteroids*<br><br>• Inhibition of *C. albicans* pseudomycelium formation (MEC)<br><br>• *In vivo* survival studies in mice infected with *C. albicans*	• MIC 0.8 µg/mL against *T. asteroids*<br><br>• MEC 0.31 µg/mL higher compared to ketoconazole<br><br>• 100% of mouse survival after 7 days at dose of 50 mg/kg
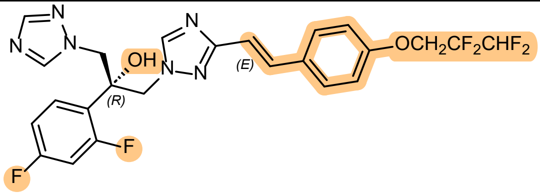<br>1992 [11]	•*In vitro* activity against *C. albicans, C. tropicalis, C. glabrata, C. neoformans, T. mentagrophytes, A. fumigatus*<br><br>•*In vivo* activity against *C. albicans, C. neoformans, A. fumigatus* in murine model<br><br>• *In vivo* toxicity parameters in primate model Compared to racemic mixture	•MIC in the range 0.1-12.5 µg/mL (for fluconazole 25-100 µg/mL)<br><br>• *C. albicans* ED50: 2.6 mg/kg (fluconazole ED50 >30)<br>• *C. neoformans* ED50: 19.7 mg/kg (fluconazole ED50 >100)<br><br>• *A. fumingatus* ED50: 39.8 mg/kg (fluconazole ED50 >57.9)<br><br>• Toxic effects at dose of 30 mg/kg, less toxic than racemic mixture

Supplementary Materials

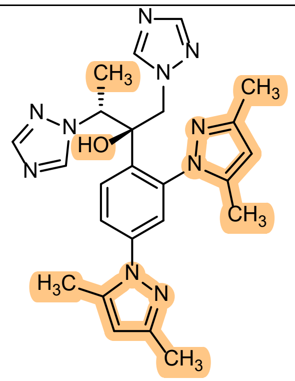<br>1993 [12]	• *In vitro* diameter of the growth inhibition zone against *C. albicans* IFO 0583	• 20 mm diameter of inhibition zone
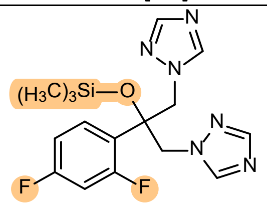<br>1995 [13]	• *In vitro* antifungal activity against *C. albicans*	• Compound is significantly more active than fluconazole, MIC 150 μg/mL (fluconazole MIC 2500 μg/mL)
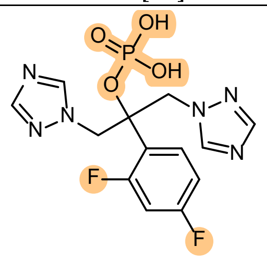<br>1997 [14]	• Solubility in water	• Significantly higher than fluconazole solubility of >150 mg/ml whereas for fluconazole 2 mg/ml
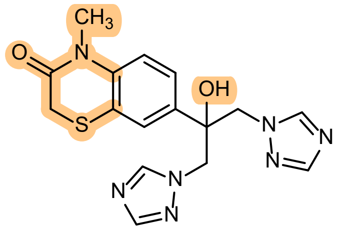<br>1998 [15]	• In vitro activity against *C. albicans*<br><br>• *In vivo* MST in mice<br><br>• CFU values for the livers of infected mice	• Activity lower than fluconazole<br>• MIC > 250 μg/mL (fluconazole <1 μg/mL)<br><br>• MST 23 days (fluconazole >60)<br><br>• CFU 49.6 (fluconazole 0)
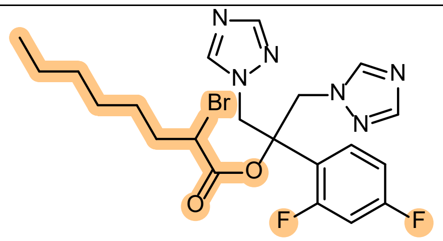<br>2004 [16]	• *In vitro* antifungal activity in RPMI and SDB medium.	• Activity lower than fluconazole in SDB medium<br><br>• MIC in RPMI *C. albicans* 111 μg/mL wheras for fluconazole MIC ≥ 4444 μg/mL.

Supplementary Materials

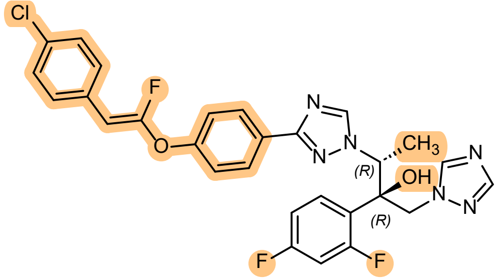<br>2005 [17]	• *In vitro* antifungal activity<br><br>• *In vivo* toxicity parameters in murine model	• Activity lower than fluconazole<br><br>• LD_{50} 1750 mg/kg
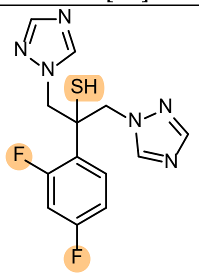<br>2005 [18]	• *In vitro* antifungal activity against *C. albicans, C. neoformans, A. niger*	• Compound was two folds more active against *C. albicans* than fluconazole in SDB medium
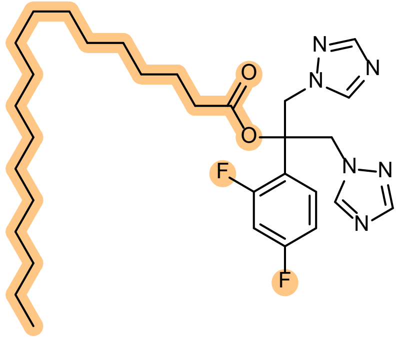<br>2007 [19]	• *In vitro* antifungal activity	• Higher activity compared to fluconazole<br><br>• pMIC (µM/mL)<br>*C. albicans* 2.54 (fluconazole 2.49)<br>*A. niger* 2.94<br>(fluconazole 2.64)
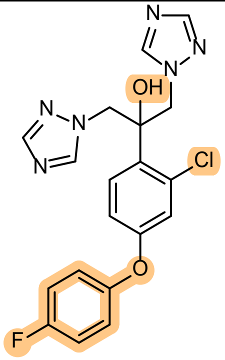<br>2009 [20]	• *In vitro* percentage inhibition of growth against fungi causing plant diseases,<br>at concentration of: 50, 5 and 1 µg/mL	• More active than terbuconazole and difenoconazole against *F. oxysporum*<br><br>• 90% inhibition growth at 1 µg /ml against *F. oxysporum*<br><br>• Activity against other tested species in range of 21.8 – 75 %

Supplementary Materials

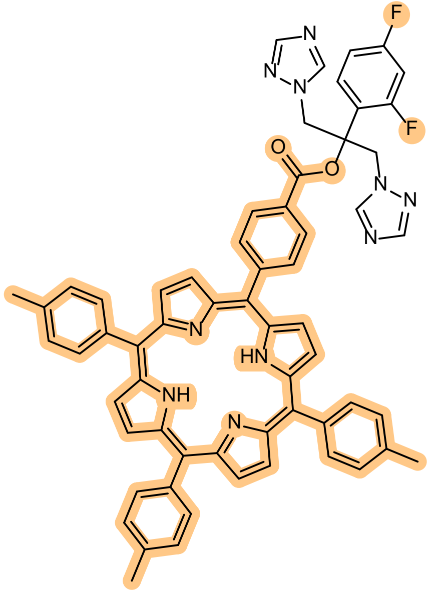<br>2010 [21]	• *In vitro* antifungal activity and light sensitivity.	• Compound activity against *C. albicans* was comparable to fluconazole without exposition to light.<br><br>• Activity increased with light exposure, this indicate light sensitivity.<br><br>• The solubility of this compound in water is lower than that of fluconazole
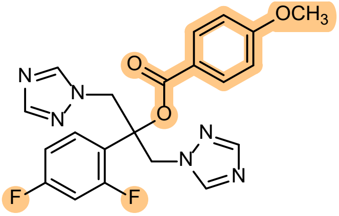<br>2010 [22]	• *In vitro* antifungal activity	• Higher activity compared to fluconazole<br><br>• MIC against *C. albicans* 1.5 µg/mL (for fluconazole 2 µg/mL)
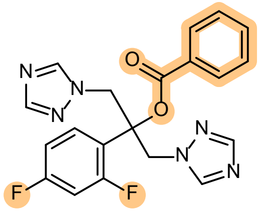<br>2010 [22]	• *In vitro* antifungal activity	• Activity comparable to fluconazole<br><br>• MIC against *C. albicans* 2 µg/mL (fluconazole 2 µg/mL)
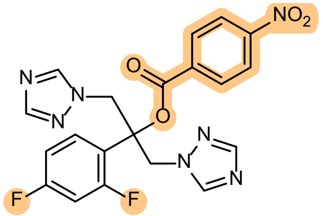<br>2010 [22]	• *In vitro* antifungal activity	• Lower activity compared to fluconazole<br><br>• MIC against *C. albicans* 25 µg/mL (fluconazole 2 µg/mL)

Supplementary Materials

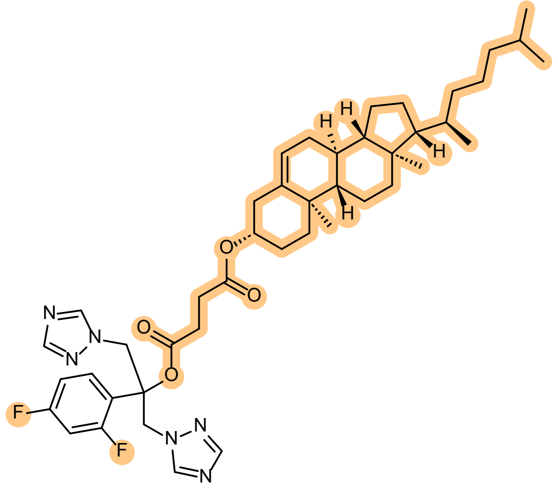 2010 [23]	• Solubility and stability in solvents used in pharmacy, water and water mixed with vitamins or polymers	• Better than fluconazole solubility and high stability in those solvents.
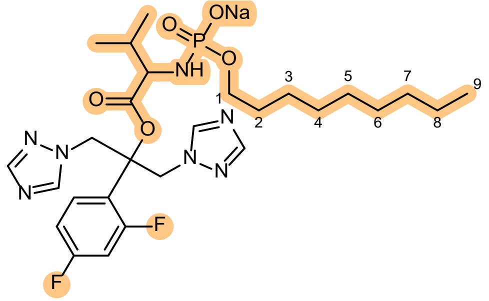 2010 [24]	• Solubility and stability in water and water with cyclodextrins.	• Better than fluconazole solubility and high stability in those solvents.
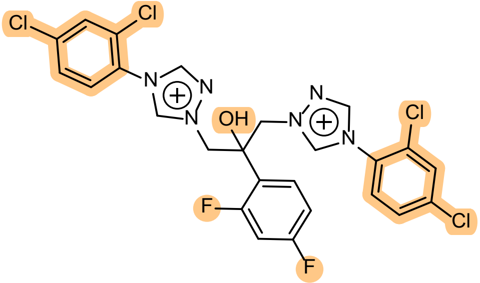 2011 [25]	• *In vitro* activity against gram positive bacteria	• Broader antibacterial spectrum compared to chloromycine<br><br>• MIC for *S. aureus, P. hauseri, E. coli* in range of 0.5-8 μg/mL
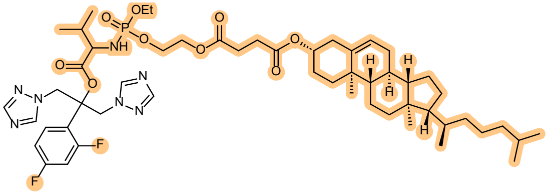 2012 [26]	• Solubility in various pharmaceutical solvents were determined	• Good solubility in less polar pharmaceutical solvents.<br><br>• Poor solubility in more polar solvents
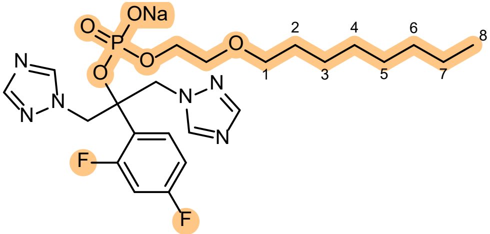 2012 [26]	• Solubility in various pharmaceutical solvents were determined	• Good solubility in both more and less polar solvents

Supplementary Materials

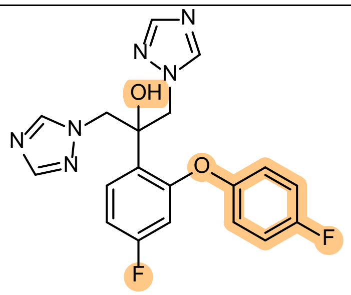<br>2015 [27]	• *In vitro* percentage inhibition of growth at a concentration of 50 µg/mL	• Low activity against tested strains<br>• *G. zeae* (44.1%)<br>• *A. solani* (20.0%)<br>• *C. archidicola* (50.0%)<br>• *F. oxysporum* (38.5%)<br>• *P. pircola* (41.2%)
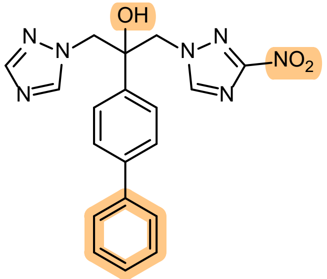<br>2015 [28]	*In vitro* activity against *T. cruzi* and *T. brucei* and toxicity against rat skeletal (L6) myoblast cell line<br><br>• *In vivo* activity in murine model<br><br>• *In vitro* ADME parameters: caco-2 permeability and metabolic stability in the presence of mouse and human microsomes	• *T. cruzi* (MIC = 0.033 µg/mL, SI = 3807.7) significantly higher activity and selectivity than fluconazole<br><br>• *T. brucei* (MIC = 2.887, SI = 44)<br><br>• Reduction of PI to >4% at 15mg/kg/day after 10 days<br><br>• High stability and permeability
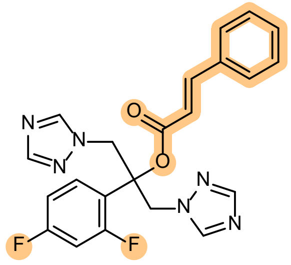<br>2016 [29]	• *In vitro* activity against biofilm formation of *C. albicans*	• $BMIC_{50}$ 4 µg/mL compared to cinnamic acid with $BMIC_{50}$ > 128 µg/mL

Supplementary Materials

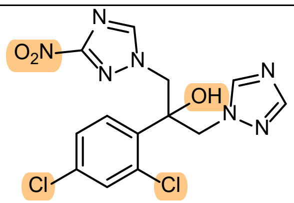<br>2017 [30]	• *In silico* molecular docking to 14α-demethylase<br><br>• *In vitro* activity against *Candida* and filamentous fungi	• FDE -11.98 Kcal/mol For fluconazole -6.74 Kcal/mol<br><br>• MIC values for *Candida* strains comparable to fluconazole<br><br>• MIC 0.5 μg/mL for clinically isolated strain of *C. albicans* (fluconazole 1 μg/mL)<br><br>• MIC value for *M. gypseum* 4 μg/mL (fluconazole 8 μg/mL)
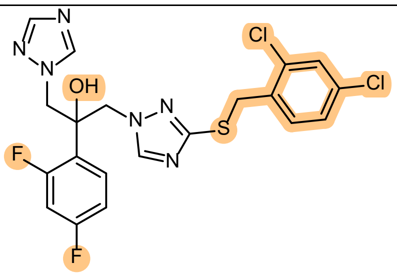<br>2018 [31]	• *In vitro* antifungal activity	• MIC value against *C. albicans* 0.125 μg/mL (fluconazole 1 μg/mL)<br><br>• MIC values against nonalbicans *Candida* species 0.063-0.5 μg/mL (fluconazole 0.5-4 μg/mL)

Supplementary Materials

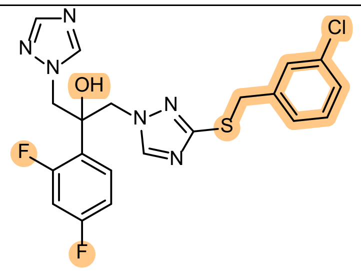<br>2018 [31]	• *In vitro* antifungal activity against fluconazole sensitive *Candida* strains<br><br>• *In vitro* antifungal activity against fluconazole resistant *Candida* strains<br><br>• *In vitro* cytotoxicity activity against human hepatoma cells Hep-G2 and mouse embryonic fibroblast cells NIH-3T3	• MIC values against nonalbicans *Candida* species 0.063-0.125 μg/mL (fluconazole 0.5-4 μg/mL)<br><br>High activity against fluconazole resistant *Candida* strains in range 0.125-16 μg/ml<br><br>• IC_{50} for Hep-G2 138 μg/ml<br>• IC for NIH-3T3 165.6 μg/ml
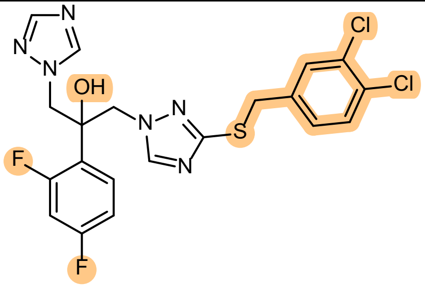<br>2018 [31]	• *In vitro* antifungal activity<br><br>• *In vitro* cytotoxicity activity against human hepatoma cells Hep-G2 and mouse embryonic fibroblast cells NIH-3T3	• High activity against fluconazole resistant *Candida* species in range 0.063-32 μg/mL<br><br>• IC_{50} for Hep-G2 83.2 μg/ml<br>• IC for NIH-3T3 145 μg/ml

Supplementary Materials

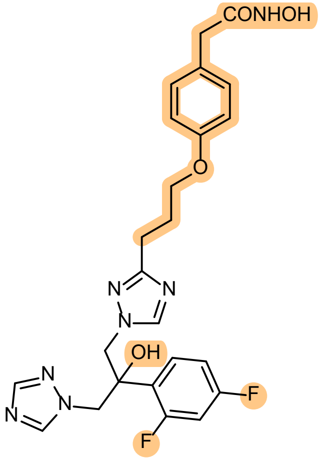<br>2020 [32]	• *In vitro* antifungal activity against Fluconazole sensitive and resistant *Candida* strains	• Some activity for *C. albicans* fluconazole resistant strains<br>• MIC in range 8-64 μg/mL<br>• No significant activity against Fluconazole sensitive *Candida* strains

1. Richardson, K. Antifungal Azole Compounds December 15, 1982, GB2099818A.

2. Rogers, T.E.; Galgiani, J.N. Activity of Fluconazole (UK 49,858) and Ketoconazole against Candida Albicans in Vitro and in Vivo. *Antimicrob Agents Chemother* **1986**, *30*, 418–422, doi:10.1128/AAC.30.3.418.

3. Worthington, P.A. Fungicidal Bis-Azolyl Compounds. January 27, 1982, EP44605.

4. Narayanaswami, S.; Richardson, K. Triazole Antifungal Agents June 6, 1983, EP96569.

5. Boyle, F.T. Antifungal Di(Azolyl)Propanol Derivatives. October 24, 1984, EP122693.

6. Richardson, K.; Narayanaswami, S. Triazole Antifungal Agents. October 17, 1984, EP122056.

7. Richardson, K.; Bass, R.J.; Cooper, K. Triazole Antifungal Agents. May 29, 1985, EP164246.

8. Boyle, F.T. Antifungal Triazole Compound. June 19, 1985, EP145314.

9. Boyle, F.T.; Gilman, D.J.; Gracvestock, M.B.; Wardleworth, J.M. Synthesis and Structure-Activity Relationships of a Novel Antifungal Agent, ICI 195,739. *Ann N Y Acad Sci* **1988**, *544*, 86–100, doi:10.1111/j.1749-6632.1988.tb40391.x.

10. Ogata, M.; Matsumoto, H.; Shimizu, S.; Kida, S.; Shiro, M.; Tawara, K. Synthesis and Oral Anti-Fungal Activity of Novel 1,3-Bis-(Azolyl)-2-Arylpropan-2-Ols. *Eur J Med Chem* **1989**, *24*, 137–143, doi:10.1016/0223-5234(89)90107-4.

11. Murakami, K.; Mochizuki, H. Optically Active Triazole Derivatives and Compositions. February 26, 1992, EP472392.

12. Itoh, K.; Okonogi, K.; Tamura, N. Optically Active Azole Compounds, Their Production and Use. June 30, 1993, EP548553.

13. Kreidl, J.; Szantay, C.; Czibula, L.; Farkas, M.; Deutsch, I.; Szegedi, M.; Hegedues, I. Novel Substituted Propane-2-Ol Derivatives March 30, 1995, WO9508552.

14. Murtiashaw, C.W.; Stephenson, P.T. Triazole Derivatives Useful in Therapy August 7, 1997, WO9728169.

Supplementary Materials

15. Fringuelli, R.; Schiaffella, F.; Bistoni, F.; Pitzurra, L.; Vecchiarelli, A. Azole Derivatives of 1,4-Benzothiazine as Antifungal Agents. *Bioorg Med Chem* **1998**, *6*, 103–108, doi:10.1016/S0968-0896(97)10016-5.

16. Nam, N.-H.; Sardari, S.; Selecky, M.; Parang, K. Carboxylic Acid and Phosphate Ester Derivatives of Fluconazole: Synthesis and Antifungal Activities. *Bioorg Med Chem* **2004**, *12*, 6255–6269, doi:10.1016/j.bmc.2004.08.049.

17. Kim, B.T.; Min, Y.K.; Lee, Y.S.; Park, N.K.; Kim, W.J. Antifungal Azole Derivatives Having a Fluorovinyl Moiety and Process for the Preparation Thereof August 9, 2005, WO2005014583.

18. Parang, K.; Sardari, S.; Nam, N.H. Azole Derivatives and Methods for Making the Same January 27, 2005, WO2005006860.

19. Ohlan, R.; Ohlan, S.; Judge, V.; Narang, R.; Ahuja, M.; Narasimhan, B. 2-(2,4-Difluorophenyl)-1,3-Bis(1,2,4-Triazol-1-Yl)Propan-2-Ol Derivatives: Synthesis, Antifungal Evaluation and QSAR Studies by Hansch Analysis. *Arkivoc* **2007**, *2007*, 172–184, doi:10.3998/ark.5550190.0008.e17.

20. Yu, G.-P.; Xu, L.-Z.; Yi, X.; Bi, W.-Z.; Zhu, Q.; Zhai, Z.-W. Synthesis and Fungicidal Evaluation of 2-Arylphenyl Ether-3-(1 *H* -1,2,4-Triazol-1-Yl)Propan-2-Ol Derivatives. *J Agric Food Chem* **2009**, *57*, 4854–4860, doi:10.1021/jf900222s.

21. Mora, S.J.; Cormick, M.P.; Milanesio, M.E.; Durantini, E.N. The Photodynamic Activity of a Novel Porphyrin Derivative Bearing a Fluconazole Structure in Different Media and against Candida Albicans. *Dyes Pigments* **2010**, *87*, 234–240, doi:10.1016/j.dyepig.2010.04.001.

22. Pawar, B.; Kanyalkar, M.; Srivastava, S. Search for Novel Antifungal Agents by Monitoring Fungal Metabolites in Presence of Synthetically Designed Fluconazole Derivatives Using NMR Spectroscopy. *Biochim Biophys Acta, Biomembr* **2010**, *1798*, 2067–2075, doi:10.1016/j.bbamem.2010.06.020.

23. Gonnissen, Y.R.J.P. Fluconazole Carboxylic Ester Derivatives, Synthesis, and Use in Long Acting Formulations September 10, 2010, WO2010100199.

24. Gonnissen, Y.R.J.P. Fluconazole Carboxylic Ester Derivatives, Synthesis, and Use in Long Acting Formulations September 10, 2010, WO2010100186.

25. Zhang, Y.-Y.; Mi, J.-L.; Zhou, C.-H.; Zhou, X.-D. Synthesis of Novel Fluconazoliums and Their Evaluation for Antibacterial and Antifungal Activities. *Eur J Med Chem* **2011**, *46*, 4391–4402, doi:10.1016/j.ejmech.2011.07.010.

26. Gonnissen, Y.R.J.P.; Voorspoels, J.F.M. Fosfluconazole Derivatives, Synthesis, and Use in Long Acting Formulations January 12, 2012, US20120010173.

27. Grammenos, W.; Boudet, N.; Mueller, B.; Escribano Cuesta, A.; Lohmann, J.K.; Grote, T.; Craig, I.R.; Fehr, M.; Quintero Palomar, M.A.; Lauterwasser, E.M.W. Preparation of Substituted [1,2,4]Triazole Compounds as Agrochemical Fungicides December 10, 2015, WO2015185708.

28. Papadopoulou, M. V.; Bloomer, W.D.; Lepesheva, G.I.; Rosenzweig, H.S.; Kaiser, M.; Aguilera-Venegas, B.; Wilkinson, S.R.; Chatelain, E.; Ioset, J.-R. Novel 3-Nitrotriazole-Based Amides and Carbinols as Bifunctional Antichagasic Agents. *J Med Chem* **2015**, *58*, 1307–1319, doi:10.1021/jm5015742.

Supplementary Materials

29. De Vita, D.; Simonetti, G.; Pandolfi, F.; Costi, R.; Di Santo, R.; D'Auria, F.D.; Scipione, L. Exploring the Anti-Biofilm Activity of Cinnamic Acid Derivatives in Candida Albicans. *Bioorg Med Chem Lett* **2016**, *26*, 5931–5935, doi:10.1016/j.bmcl.2016.10.091.

30. Sadeghpour, H.; Khabnadideh, S.; Zomorodian, K.; Pakshir, K.; Hoseinpour, K.; Javid, N.; Faghih-Mirzaei, E.; Rezaei, Z. Design, Synthesis, and Biological Activity of New Triazole and Nitro-Triazole Derivatives as Antifungal Agents. *Molecules* **2017**, *22*, 1150, doi:10.3390/molecules22071150.

31. Motahari, K.; Badali, H.; Hashemi, S.M.; Fakhim, H.; Mirzaei, H.; Vaezi, A.; Shokrzadeh, M.; Emami, S. Discovery of Benzylthio Analogs of Fluconazole as Potent Antifungal Agents. *Future Med Chem* **2018**, *10*, 987–1002, doi:10.4155/fmc-2017-0295.

32. Han, G.; Liu, N.; Li, C.; Tu, J.; Li, Z.; Sheng, C. Discovery of Novel Fungal Lanosterol 14α-Demethylase (CYP51)/Histone Deacetylase Dual Inhibitors to Treat Azole-Resistant Candidiasis. *J Med Chem* **2020**, *63*, 5341–5359, doi:10.1021/acs.jmedchem.0c00102.
